# Development of CRISPR/Cas9-Mediated Gene-Drive Construct Targeting the Phenotypic Gene in *Plutella xylostella*

**DOI:** 10.3389/fphys.2022.938621

**Published:** 2022-06-29

**Authors:** Muhammad Asad, Dan Liu, Jianwen Li, Jing Chen, Guang Yang

**Affiliations:** ^1^ State Key Laboratory of Ecological Pest Control for Fujian and Taiwan Crops, Institute of Applied Ecology, Fujian Agriculture and Forestry University, Fuzhou, China; ^2^ Joint International Research Laboratory of Ecological Pest Control, Ministry of Education, Fuzhou, China; ^3^ Key Laboratory of Integrated Pest Management for Fujian-Taiwan Crops, Ministry of Agriculture, Fuzhou, China; ^4^ Key Laboratory of Green Pest Control, Fujian Province University, Fuzhou, China; ^5^ Ministerial and Provincial Joint Innovation Centre for Safety Production of Cross-Strait Crops, Fujian Agriculture and Forestry University, Fuzhou, China

**Keywords:** homology-directed repair, non-homologous-end joining, pxyellow, gene-drive efficiency, resistant-allele formation, diamondback moth

## Abstract

The gene-drive system can ensure that desirable traits are transmitted to the progeny more than the normal Mendelian segregation. The clustered regularly interspersed palindromic repeats (CRISPR)/CRISPR-associated protein 9 (Cas9) mediated gene-drive system has been demonstrated in dipteran insect species, including *Drosophila* and *Anopheles*, not yet in other insect species. Here, we have developed a single CRISPR/Cas9-mediated gene-drive construct for *Plutella xylostella,* a highly-destructive lepidopteran pest of cruciferous crops. The gene-drive construct was developed containing a Cas9 gene, a marker gene (*EGFP*) and a gRNA sequence targeting the phenotypic marker gene (*Pxyellow*) and site-specifically inserted into the *P. xylostella* genome. This homing-based gene-drive copied ∼12 kb of a fragment containing *Cas9* gene, gRNA, and *EGFP* gene along with their promoters to the target site. Overall, 6.67%–12.59% gene-drive efficiency due to homology-directed repair (HDR), and 80.93%–86.77% resistant-allele formation due to non-homologous-end joining (NHEJ) were observed. Furthermore, the transgenic progeny derived from male parents showed a higher gene-drive efficiency compared with transgenic progeny derived from female parents. This study demonstrates the feasibility of the CRISPR/Cas9-mediated gene-drive construct in *P. xylostella* that inherits the desired traits to the progeny. The finding of this study provides a foundation to develop an effective CRISPR/Cas9-mediated gene-drive system for pest control.

## Introduction

During the Mendelian segregation, alleles generally have an equal chance (50:50) of being transmitted to progeny. Gene drives are selfish genetic elements that promote the spread of desirable traits across the populations by assuring that they are more often inherited than the Mendelian segregation (Akbari et al., 2015; Kandul et al., 2020). There are many examples of selfish genetic elements, either naturally occurring or synthetic, that can bypass the Mendelian segregation (Werren et al., 1988; Werren, 2011). The meiotic drive, sex-ratio distortion, and replicative transposition are typical examples of naturally occurring gene-drive elements (Ågren, 2016). The synthetic *Medea* drive system (Chen et al., 2007; Akbari et al., 2014), engineered underdominance systems (Buchman et al., 2018), and homing endonuclease gene-drive (HEGD) (Marshall and Hay, 2012; Esvelt et al., 2014; Gantz and Bier, 2015; Gantz et al., 2015; Champer et al., 2016) are included in synthetic gene-drive systems. The development of HEGD is accelerated by discovering the CRISPR/Cas9 system (Jinek et al., 2012; Cong et al., 2013; Mali et al., 2013).

Theoretical and practical use of CRISPR/Cas9 systems as a HEGD to change the genotype of insect species has been demonstrated (Esvelt et al., 2014; Unckless et al., 2015), which can potentially transmit the desired phenotype into a wild-type population of target species (Deredec et al., 2008; Unckless et al., 2017). In the CRISPR/Cas9-based gene drive (CCGD), the CRISPR/Cas9 construct contains the coding sequence of Cas9 endonuclease and a 20-bp gRNA targeting the specific site of the host genome. RNA-guided Cas9 causes a break of double-stranded DNA in the wild-type allele, which is repaired through either HDR or NHEJ (Karaminejadranjbar et al., 2018). The HDR-mediated repair drives the desired components into the next generation, while NHEJ mediated repair leads to the formation of resistant allele without transferring the desirable traits into the next generation (Champer et al., 2017; Champer et al., 2018). The consequences of resistant alleles depend on several factors, including the fitness cost associated with the drive and the type of gene-drive approach.

The CCGD system has been successfully developed in different organisms including bacteria (Valderrama et al., 2019), yeast (Dicarlo et al., 2015; Roggenkamp et al., 2018; Shapiro et al., 2018), mammals (Grunwald et al., 2019), and insects (Gantz and Bier, 2015; Gantz et al., 2015; Champer et al., 2017; Karaminejadranjbar et al., 2018; Kyrou et al., 2018; Champer et al., 2020; Li et al., 2020). These studies show extremely-variable gene-drive efficiency, close to 100% in *Saccharomyces cerevisiae*, 19%–62% in *Drosophila melanogaster* and 87%–99% in *Anopheles*. The variability in gene-drive efficiency depends on the timing and level of Cas9 expression, organism-specific factors, and the genomic targets (Champer et al., 2019). The CCGD has recently been used to control the pest population through a population-modification and population-suppression drives. In a population-modification drive, the release of transgenic mosquitoes in which the CCGD contains effector genes inhibiting the mosquito pathogen transmission results in the replacement of disease-sensitive mosquitoes with disease-resistant mosquitoes, which decreases the pathogen transmission (Buchman et al., 2018; Buchman et al., 2020). In the population-suppression drive, the homing-based gene-drive is developed to target the conserved sex-specific genes (Kyrou et al., 2018). Given these characteristics, both alteration and suppression drives can be used to control the pest population.

The germline-specific promoters and phenotypic marker genes play a significant role in the development of CCGD. The germline-specific promoters, including *nanos* promoter and *vasa* promoter to drive germline-specific *Cas9* expression, have shown great potential to develop the CCGD in insects (Gantz and Bier, 2015; Gantz et al., 2015; Champer et al., 2017; Karaminejadranjbar et al., 2018; Kyrou et al., 2018; Champer et al., 2020; Li et al., 2020). Similarly, the phenotypic marker genes such as *yellow* and *white* are used as the target sites to assess and minimize the fitness cost caused by the CCGD. Furthermore, the phenotypic genes also provide an easy way to screen the progeny with resistant alleles through NHEJ (Gantz and Bier, 2015; Gantz et al., 2015; Champer et al., 2017; Champer et al., 2018). Therefore, we selected *PxnanosO* promoter to drive *Cas9* and *yellow* gene as the target site for CCGD.

The diamondback moth (*Plutella xylostella*) is a globally-distributed lepidopteran pest that mainly attacks cruciferous crops and has developed resistance to all classes of insecticides, making it difficult to control (Furlong et al., 2013). Genetic-based approaches, especially CCGD for population suppression and modification, have only been developed in dipteran insect species (Gantz and Bier, 2015; Gantz et al., 2015; Champer et al., 2017; Karaminejadranjbar et al., 2018; Kyrou et al., 2018; Li et al., 2020), not yet in other insect species. Previously, a CRISPR/Cas9-based split drive system has been successfully developed in *Plutella xylostella*. However, this split drive system failed to produce homing-based progeny (Xu et al., 2022). No homing-based progeny in the split-drive system might be because the two different transgenic lines (gRNA line and Cas9 line) cross each other. Keeping in view of these results, we developed a single CCGD construct for the first time in *P. xylostella* and evaluated its efficiency. The results provide a foundation for developing a CCGD system for population suppression or modification of *P. xylostella*.

## Materials and Methods

### Rearing of *P. xylostella* Strain

The insecticide-susceptible strain Geneva 88 (G88) of *P. xylostella* used in this study was obtained from Cornell University in 2016 and subsequently established as a colony at the Institute of Applied Ecology, Fujian Agriculture and Forestry University. This strain was reared using a prepared artificial diet at 35%–50% RH, 16 h: 8 h (L:D) photoperiod, and 25 C in the growth chamber. After the larvae developed into pupae, the pupae were collected and transferred into the box for eclosion and mating. The adults were kept at 25 C and 80% RH and fed with 10% honey solution.

### Amplification and Cloning of *Pxyellow* Gene Target Site

The genomic DNA of fourth instar larvae of *P. xylostella* was extracted using the MEGA Bio-Tek Tissue DNA kit (Omega, Norcross, United States), followed by purification after RNase treatment. The sequence of the candidate *Pxyellow* gene was obtained from *P. xylostella* genomic database (http://iae.fafu.edu.cn/DBM/index.php). The specific primers for PCR amplification were designed by using the NCBI database’s primer tool (https://www.ncbi.nlm.nih.gov/tools/primer-blast/). The 2.3-kb fragment of the *Pxyellow* gene, including the gRNA target site, was PCR amplified with designed primer Yts-F and Yts-R (Supplementary Table S1). The PCR reaction was prepared by using the Max Super-Fidelity DNA Polymerase (Vazyme, Nanjing, China) and carried out with the following conditions of 95 C for 3 min; 32 cycles of 95 C for 10 s, 62 C for 20 s, 72 C for 2 min; then 72 C for 5 min; and 4 C forever. The PCR amplified products were purified with the Omega Gel Purification Kit (Omega, Norcross, United States) following its protocol. The purified PCR product was sub-cloned into the PJET1.2 blunt-end vector (Thermo Scientific, Waltham, MA, United States) and confirmed through Sanger sequencing.

### Design and *in vitro* Transcription of sgRNA

The DNA fragment at exon 3 of *Pxyellow* gene was selected as the target site of gRNA based on the 5′-GG-(N)18-NGG-3′ principle (Hwang et al., 2013) by using the CRISPR gRNA Design tool-ATUM (https://www.atum.bio/eCommerce/cas9/input). The GG bases were added at the 5′ end of the gRNA target site to ensure the *in vitro* transcription stability by T7 RNA polymerase. A pair of long oligonucleotides were used to produce a DNA template of sgRNA for *in vitro* transcription (Bassett et al., 2013). The sgRNA-F oligonucleotide contained a T7 RNA polymerase binding site and the sgRNA target site, and the sgRNA-R oligonucleotide contained gRNA scaffold and overlap region of forward primer (Supplementary Table S1). PCR reaction was carried out by using the PrimeSTAR HS DNA Polymerase (TaKara Biomedical Technology, Beijing, China) at the following conditions: 98 C for 3 min; 32 cycles of 98 C for 10 s, 55 C for 20 s, 72 C for 30 s; then 72 C for 5 min and 4 C forever. This PCR product was purified with the Omega universal DNA Purification Kit (Omega, Norcross, United States) by following its protocol. The purified PCR product was used for *in vitro* transcription of sgRNA with the HiScribe T7 Quick High Yield RNA Synthesis Kit (New England Biolabs, Ipswich, United States) by following its protocol.

### Construction of PJET*-*Cas9 Cassette

Firstly, we obtained the germline-specific *nanos* gene sequence of *Bombyx mori* (NP_001093314) (Xu et al., 2019) from the NCBI (National Center for Biotechnology Information) (https://www.ncbi.nlm.nih.gov/) and blasted it against the *P. xylostella* genomic database (http://iae.fafu.edu.cn/DBM/index.php) (You et al., 2013). The highly-similar gene *PxnanosO* (gene id: Px008918) was identified and selected. The *PxnanosO* promoter (PxnanosP) was amplified by using Nanos-F and Nanos-R primers (Supplementary Table S1). The PCR reaction was prepared by using the Super-Fidelity DNA Polymerase (Vazyme, China) and carried out with the following PCR conditions: 95 C for 3 min; 32 cycles of 95 C for 15 s, 60 C for 20 s, 72 C for 1 min; 72 C for 5 min; and then 4 C forever. This amplified PCR product was further purified with the Omega Gel Purification Kit (Omega, Norcross, United States). The purified PCR product was sub-cloned into the PJET1.2 blunt-end vector (ThermoFisher Scientific, Waltham, MA, United States) and confirmed through sequencing. After sequence confirmation, we amplified PxnanosP from the PJET1.2 blunt-end vector with the same primers and conditions mentioned above.

The 4-kb Cas9 fragment was amplified from the plasmid PTD-T7-Cas9 (Huang et al., 2016) by using a pair of primers of Cas9-F and Cas9-R (SI, Supplementary Table S1) with the overlapping region of PxnanosP and SV40 PolyA tail*.* The PCR reaction was carried out with the Super-Fidelity DNA Polymerase (Vazyme, China) at the following conditions: 95 C for 3 min; 32 cycles of 95 C for 15 s, 58 C for 15 s, 72 C for 3 min; 72 C for 10 min; and then 4 C forever. The 4-kb amplified Cas9 product was further purified with the Omega Gel Purification Kit (Omega, Norcross, United States).

The 250-bp Sv40 PolyA tail fragment was amplified from the GPXL-BacII-IE1-EGFP-SV40 vector (Asad et al., 2020) by using primers of Sv40-F and Sv40-R (Supplementary Table S1) with the overlapping region of Cas9 fragment and PJET1.2 vector. The PCR reaction was carried out with the Super-Fidelity DNA Polymerase (Vazyme, China) at the following conditions: 95 C for 3 min; 32 cycles of 95 C for 15 s, 58 C for 15 s, 72 C for 3 min; 72 C for 10 min; and then 4 C forever. The PCR amplified product of 250-bp Sv40 PolyA tail was further purified with the Omega Gel Purification Kit.

These three fragments were assembled and cloned into the PJET1.2 blunt-end vector by using the HiFi DNA Assembly Master Mix (New England Biolabs, #E5510) by following its protocol to obtain the PJET-Cas9 vector (Supplementary Figure S1).

All oligonucleotides containing overlapping regions for assembling fragments into vectors were designed by using the NEB Builder Assembling Tool of New England Bio Lab (http://nebuilder.neb.com/). The vector and insert fragment concentrations were calculated by using the New England Bio Lab tool NEBio Calculator (https://nebiocalculator.neb.com/#!/ligation). The mixture concentrations of all digestion reactions were calculated by using the New England Bio Lab tool, NEB Cloner (http://nebcloner.neb.com/#!/).

### Insertion of Hr5IE1-EGFP-Sv40 Fragment to PJET-Cas9 Vector

The Hr5IE1-EGFP-Sv40 fragment was amplified from the previously constructed vector GPXL-BacII-IE1-EGFP-SV40 (Asad et al., 2020). Two oligonucleotides (IE1-F and IE1-R) were used to amplify the Hr5IE1-EGFP fragment (Supplementary Table S1), which contains an overlapping region for insertion of this fragment at *Abs*Ⅰ cutting site of PJET-Cas9 vector (Supplementary Figure S1). The PCR reaction was carried out with the Super-Fidelity DNA Polymerase (Vazyme, China) at the following conditions: 95 C for 3 min; 30 cycles of 95 C for 30 s, 58 C for 30 s, 72 C for 2 min; 72 C for 5 min and then 4 C forever. The amplified product of the Hr5IE1-EGFP-Sv40 fragment was further purified with the Omega Gel Purification Kit (Omega, Norcross, United States). The PJET-Cas9 vector was linearized with the digestion of *Abs*Ⅰ (SibEnzyme, Russia). This digested product was purified by using the Omega Gel Purification Kit (Omega, Norcross, United States). These two purified fragments (Hr5IE1-EGFP-Sv40 fragment and linearized PJET-Cas9 vector) were assembled with the HiFi DNA Assembly Master Mix (New England Biolabs, Ipswich, United States) by following the instruction of the manufacturer to obtain the PJET-Cas9-EGFP vector (Supplementary Figure S2). The insertion of Hr5IE1-EGFP-Sv40 fragment into the PJET-Cas9 vector was confirmed through colony PCR. The plasmid of positive clones was extracted with the Omega Mini Plasmid Extraction Kit (Omega, Norcross, United States) by following its protocol. The extracted plasmids of PJET-Cas9-EGFP vector were further used in double digestion with HF *Not*I (New England Biolabs, Ipswich, United States) and HF *Age*I (New England Biolabs, Ipswich, United States).

### Insertion of PxU6-gRNA Cassette to PJET-Cas9-EGFP Vector

The PxU6 promoter was amplified from the previously-used U6:sgRNA expression vector (Huang et al., 2017). To link the gRNA sequence with U6, 20-bp gRNA and a sequence of terminal signals for the *PxU6* gene were added to the reverse primer U6-R (Supplementary Table S1). The PCR reaction mixture was prepared with the Super-Fidelity DNA Polymerase (Vazyme, China) by using two designed primers (U6-F and U6-R). The PCR reaction was carried out with following conditions: 95 C for 4 min; 35 cycles of 95 C for 30 s, 61 C for 30 s, 72 C for 1 min; 72 C for 5 min and then 4 C forever. Another PCR reaction was performed with two oligonucleotides (gRNA-F and gRNA-R) containing the overlapping sequence for insertion of this fragment to the PJET-Cas9-EGEP vector. The PCR reaction was carried out with the Super-Fidelity DNA Polymerase (Vazyme, China) at the same conditions used to amplify PxU6 promoter. This amplified product was further purified with the Omega Gel Purification Kit (Omega, Norcross, United States). The PJET-Cas9-EGFP vector was digested with *Age*I (New England Biolabs). The digested product was purified with the Omega Gel Purification Kit (Omega, Norcross, United States). The two fragments (PJET-Cas9-EGFP vector and PxU6*-*gRNA) were assembled with the HiFi DNA Assembly Master Mix (New England Biolabs, #E5510) by following the manufacturer’s instruction to obtain the PJET-Cas9-EGFP-gRNA vector (Supplementary Figure S3). The plasmid of positive clones was extracted with the Omega Mini Plasmid Extraction Kit (Omega, Norcross, United States). The extracted plasmids were further used in double digestion with *Spe*I and *Age*I enzymes (New England Biolabs, Ipswich, United States).

### Cloning of Homologous Arms

The 1-kb left and right regions from the gRNA target site of *Pxyellow* gene were selected as homologous arms. These two homologous arms were PCR amplified from the cloned *Pxyellow* gene with a pair of primers (LH-F & LH-R, RH-F & RH-R). The PCR reactions were carried out with the Super-Fidelity DNA Polymerase (Vazyme, China) at the following conditions: 95 C for 3 min; 35 cycles of 95 C for 15 s, 61 and 58 C for 30 s, 72 C for 1 min; 72 C for 5 min and then 4 C forever. These two amplified products were purified by gel electrophoresis and separately ligated into the PJET1.2 blunt vector. The ligated vector was confirmed through Sanger sequencing.

### Insertion of Left and Right Homologous Arms to PJET-Cas9-EGFP-gRNA Vector

The left homologous arm (LHA) was PCR amplified by using the cloned fragment with two oligonucleotides (LHA-F and LHA-R) containing overlapping regions for assembling with the PJET-Cas9-EGFP-gRNA vector (Supplementary Table S1). The PCR reaction was carried out with the Super-Fidelity DNA Polymerase (Vazyme, China) at the following conditions: 95 C for 3 min; 35 cycles of 95 C for 15 s, 58 C for 30 s, 72 C for 1 min; 72 C for 5 min and then 4 C forever. The PJET-Cas9-EGFP-gRNA vector was digested with *Spe*I and then purified with the Omega Gel Purification Kit (Omega, Norcross, United States). The digested vector PJET-Cas9-EGFP-gRNA and amplified left homologous arm were assembled with the HiFi DNA Assembly Master Mix (New England Biolabs, #E5510). The insertion of LHA into PJET-Cas9-EGFP-gRNA was confirmed through colony PCR. The plasmid of positive clones was extracted with the Omega Mini Plasmid Extraction Kit (Omega, Norcross, United States)

The confirmed vector containing LHA was further digested with the *Age*I (New England Biolabs, Ipswich, United States) followed by purification with the Omega Gel Purification Kit (Omega, Norcross, United States). The right homologous arm (RHA) was PCR amplified by using the cloned fragment with two oligonucleotides (RHA-F and RHA-R) containing overlapping regions for assembling with the digested vector (Supplementary Table S1). The PCR reaction was carried out with the Super-Fidelity DNA Polymerase (Vazyme, China) at the following conditions: 95 C for 3 min; 32 cycles of 95 C for 15 s, 60 C for 15 s, 72 C for 1 min; 72 C for 5 min and then 4 C forever. The right homologous arm and digested vector were again assembled with the HiFi DNA Assembly Master Mix (New England Biolabs, #E5510) to obtain the PJET-Ca9-EGFP-gRNA-LRH vector (SI Supplementary Figure S4). The insertion of RHA was confirmed through colony PCR. The final gene-drive construct was named LHA-Ca9-EGFP-gRNA-RHA. The plasmid of positive clones was extracted with the Omega Mini Plasmid Extraction kit (Omega, Norcross, United States). The extracted plasmids were digested with HF *Not*I (New England Biolabs, Ipswich, United States) and HF *Age*Ⅰ (New England Biolabs, Ipswich, United States) to confirm the insertion of LHA and RHA fragments into the PJET-Cas9-EGFP-gRNA vector.

### Generation of Double-Stranded RNA to Target *Pxku70*

The putative sequence of *Pxku70* gene was obtained by blasting the X-ray repair protein 5-like mRNA (LOC101736121) sequence of *Bombyx mori* in the DBM genomic database (http://iae.fafu.edu.cn/DBM/index.php). The primers (Ku70-F and Ku70-R) were designed to amplify 610-bp fragment based on the obtained sequence (Supplementary Table S1). PCR reaction was carried out with the Super-Fidelity DNA Polymerase (Vazyme, China) at the following conditions: 95 C for 3 min; 28 cycles of 95 C for 15 s, 60 C for 15 s, 72 C for 1 min; 72 C for 5 min and then 4 C forever. Furthermore, a pair of primers of T7Ku70-F and T7Ku70-R were designed to contain a T7 promoter in both primers (Supplementary Table S1). The PCR reaction was carried out with the Super-Fidelity DNA Polymerase (Vazyme, China) by using the previously-amplified product as the template at the same conditions explained above. The amplified product was used as the template for dsRNA synthesis with the T7 RiboMAX™ Express RNAi Kit (Promega) by following its protocol.

### Microinjection

The injection mixture was prepared by mixing Cas9-N-NLS Nuclease (GenScript, United States), gRNA targeting *Pxyellow*, dsRNA targeting *Pxku70,* LHA-Cas9-EGFP-gRNA-RHA vector with the injection buffer as previously described (Asad et al., 2020). The parafilm sheets of 12 cm^2^ coated with the cabbage leave extract were used to collect eggs, and the sheets were replaced every 30 min by new ones during the oviposition of female adults. The injections were performed within 1 h after oviposition by using the Olympus SZX16 microinjection system (Olympus, Japan). The injected embryos were placed in the hatching chamber at 25 ± 1 C, 60 ± 10% RH.

### Crossing and Screening

The hatched larvae from the injected eggs were maintained on the freshly-prepared diet. The G_0_ females and males were separated and outcrossed with wild-type adults of 1 G_0_ female with 2 wild-type males and 1 G_0_ male with 2 wild-type females. However, An equal number of transgenic and wild-type individuals (one transgenic cross with one wild-type individual) were used for the subsequent crosses of F_2_, F_3,_ and F_4_ generations. The larval progeny of F_1_, F_2_, F_3,_ and F_4_ were screened by the EGFP fluorescence under the fluorescent microscope equipped with an EGFP filter and stereoscope. The homozygous larvae exhibited a strong EGFP signal, and the heterozygous larvae showed a patchy EGFP signal (Chen et al., 2021).

### Confirmation of Gene Drive

The genomic DNA was extracted from adults of F_1_ generation after oviposition with the Omega Genomic DNA Extraction Kit (Omega, Norcross, United States). A pair of primers (gTS-F and gTS-R) were used to amplify the gRNA target site (Supplementary Table S1). The amplified products were confirmed through Sanger sequencing. The insertion of the gene-drive cassette into the *Pxyellow* gene was confirmed through PCR by using the designed primers (Supplementary Table S1). PCR reaction was carried out with the Super-Fidelity DNA Polymerase (Vazyme, China) and the extracted DNA of F_1_ EGFP-positive adults at the conditions: 95 C for 3 min; 32 cycles of 95 C for 15 s, 60 C for 15 s, 72 C for 1 min; 72 C for 5 min and then 4 C forever. The amplified products were confirmed through Sanger sequencing.

### Statistical Analysis

The Chi-square (*χ* ^2^) analysis was performed by using the Graphpad 8.02 (GraphPad Software, La Jolla, San Diego, CA, United States) to determine the significant difference between the gene-drive efficiency in different generations of transgenic *P. xylostella* and EGFP^+^ progeny derived from different parents at *p* < 0.05. The *Z* test was used to compare the NHEJ and HDR mutation rates in the progeny of different crosses at *p* < 0.05.

## Results

### Confirmation of the Target Site *Pxyellow* Gene

**FIGURE 1:**
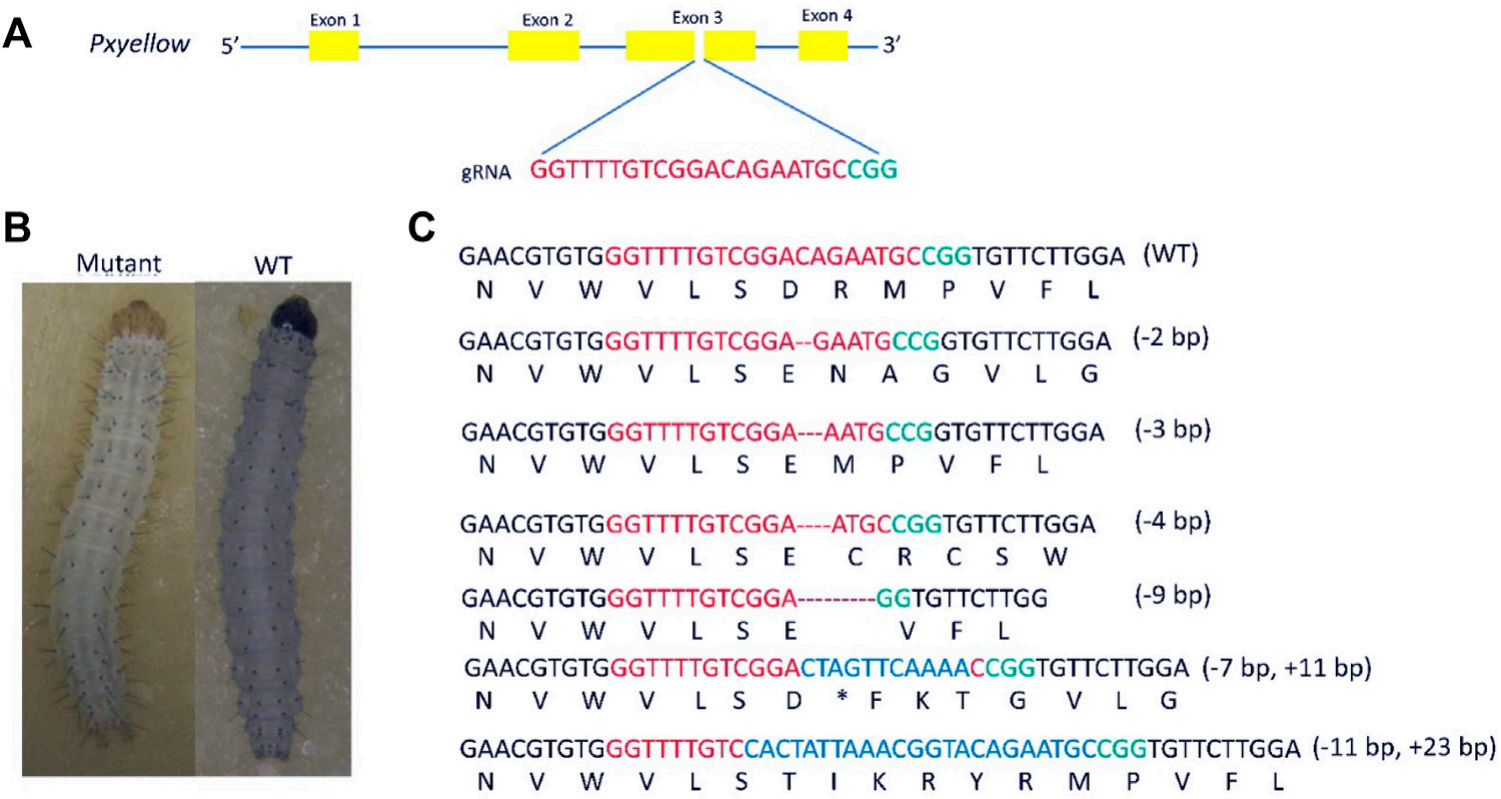
Knockout of *Pxyellow* gene at the target site. **(A)**, the schematic representation of *Pxyellow* gene. The orange boxes indicate the exon of *Pxyello*w gene, and blue lines indicate the introns. **(B)**, phenotypic difference between *Pxyellow* mutant and wild type. **(C)**, types of deletions and insertions at the target site. Red base pairs indicate the gRNA sequence and green indicate the PAM, sequence; the deletions are highlighted with dashes (--), and the inserted base pairs are highlighted with blue colors.

The *Pxyellow* gene (gene ID: *Px007091*) is body pigmentation gene and disruption of *Pxyellow* gene only leads to a change in body color (Wang et al., 2020). Therefore, the *Pxyellow* gene is a suitable target site for screening the mutant progeny in the CRISPR/Cas9-based gene-drive system in *P. xylostella*. The *Pxyellow* gene is located at scaffold 25, containing four exons and five introns. The sgRNA was designed and synthesized to target the Exon 3 of *Pxyellow* (Figure 1A). A total of 150 preblastoderm embryos were injected with the mixture of sgRNA and Cas9 protein, and 90 hatched (the hatching rate of 60%). The 60 G_0_ larvae showed yellow head compared with wild-type larvae, which was clearly observed at second and third instar larvae (Figure 1B), making the mutation rate 40% (60/150) in G_0_ individuals. The 439-bp DNA fragment flanking the target site was amplified from these mosaic G_0_ adults and sequenced.

Out of these 60 G_0_ mutant individuals, 43 individuals showed indels at the target site (Supplementary Appendix Table S1). Four types of deletions (-2 bp, -4 bp. -3 bp and -9 bp) and two types of insertions (+11 bp and +14 bp) were observed in 43 individuals (Figure 1C).

### Construct Design and Development of Transgenic *P. xylostella*

**FIGURE 2:**
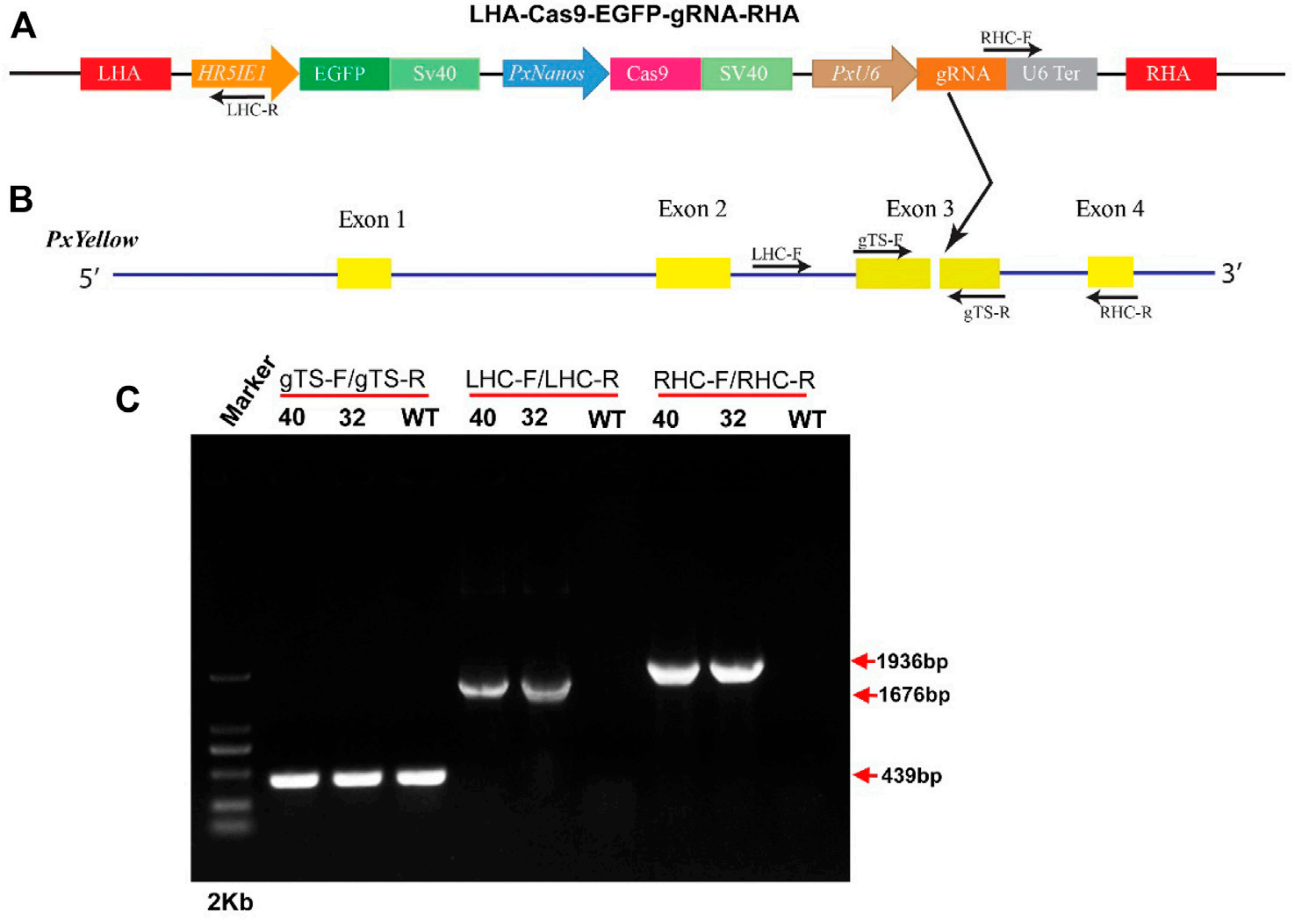
Site-specific insertion of gene-drive construct. **(A)**, schematic representation of gene-drive cassette (LHA-Cas9-EGFP-gRNA-RHA). **(B)**, schematic representation of *Pxyellow* gene. The small arrow with the label represents the position, orientation and name of primers used to confirm the integration of the gene-drive cassette into the *Pxyellow* target site. The red boxes represent the left homologous arm (LHA) and right homologous arm (RHA). The other components of plasmid LHA-Cas9-EGFP-gRNA-RHA have been highlighted and labeled with different colors. The yellow boxes represent the 4 exons of *Pxyellow* gene. The dark gray lines represent the introns of *Pxyellow* gene. The black arrow represents the gRNA target site in *Pxyellow* locus. **(C)**, Verification of the site-specific insertion of LHA-Cas9-EGFP-gRNA-RHA construct by PCR.

The 1.8-kb upstream sequence of the *Pxnanos*O gene was cloned and used as a putative promoter. The immediate-early-stage 1 promoter with an enhancer 5 (HR5IE1) was used to drive the EGFP marker gene. This promoter has been successfully used as a transformation marker in different insect species, including *P. xylostella* (Grossman et al., 2001; Gong et al., 2005; Martins et al., 2012). The PxU6:3 promoter has been used to drive gRNA in *P. xylostella* (Huang et al., 2017). The LHA-Cas9-EGFP-gRNA-RHA construct contained a *Cas9* gene driven by Pxnanos promoter, an *EGFP* marker gene-driven by HR5IE1 promoter and a gRNA sequence (targeting the phenotypic marker gene *Pxyellow*) driven by PxU6 promoter (Figure 2A). The details about the construction and verification of LHA-Cas9-EGFP-gRNA-RHA construct are listed in Supplementary information (Supplementary Figure S5). The detailed sequences of Pxnanos promoter, HR5IE1 promoter, *EGFP* marker gene, Cas9, PxU6 promoter gRNA, left and right homologous arms are also listed in (Supplementary Appendix Table S2).

**FIGURE 3:**
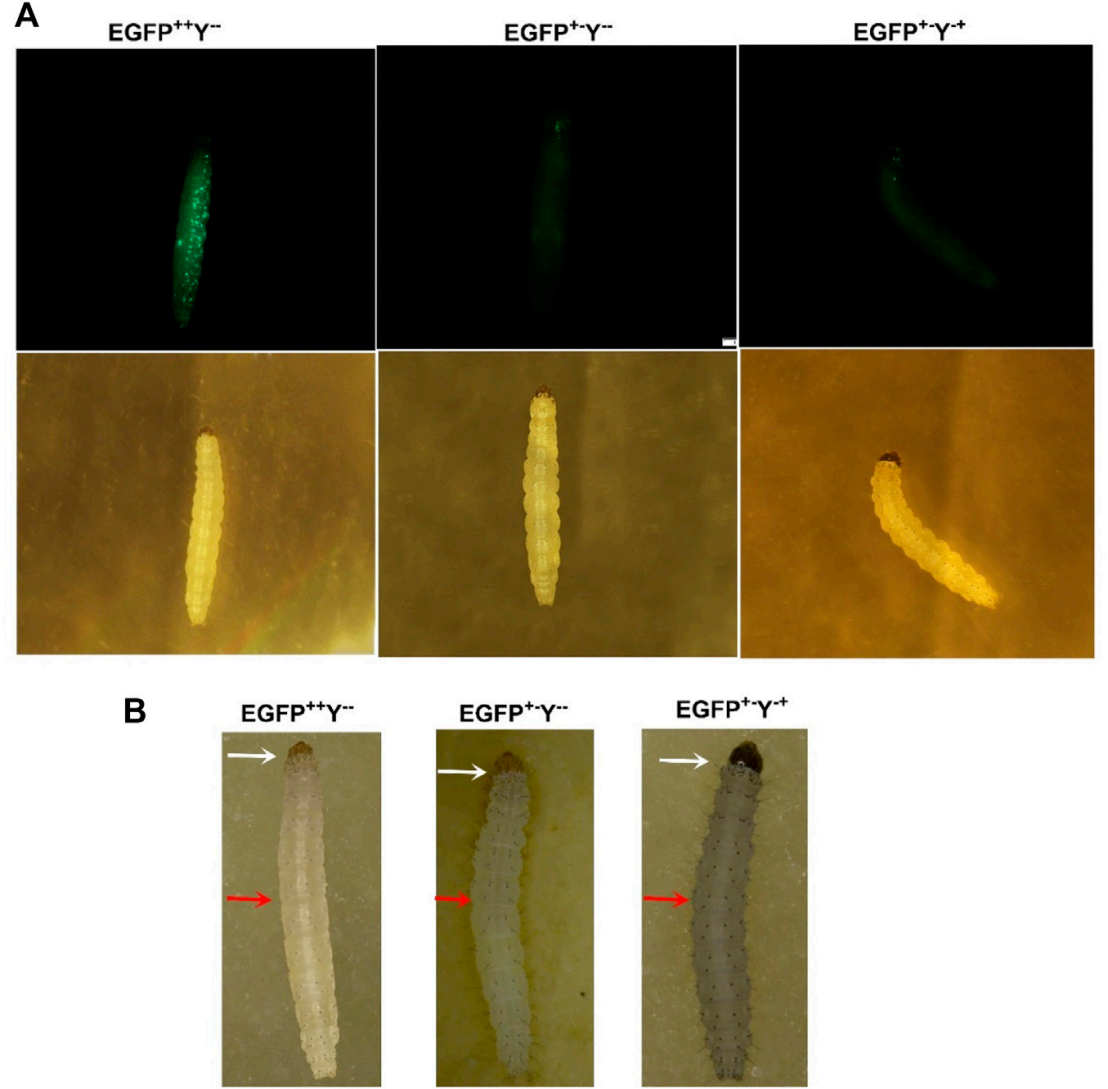
Larval phenotypes of transgenic *P. xylostella* harboring the gene-drive cassette. **(A)**, larval images in the upper row taken in the presence of EGFP filter and larval images in the lower row taken with a bright-light filter. **(B)**, larval images taken by a stereoscope. The white arrows indicate the head color, and the red arrows indicate the body color.

The transgenic *P. xylostella* was developed by injecting the mixture of plasmid LHA-Cas9-EGFP-gRNA-RHA, Cas9 protein, sgRNA, and *Pxku70* dsRNA into embryos to insert the gene-drive cassette Cas9-EGFP-gRNA into the target site of *Pxyellow* (Figure 2B). A total of 5930 *P. xylostella* embryos were injected in five attempts, of which the mean G_0_ survival rate was 22.02% (Supplementary Table S2). The survived G_0_ male and female were individually outcrossed with wild-type to produce the F_1_ progeny. The integration of drive cassette in F_1_ was verified by the PCR products a 439-bp fragment amplified with primers gTS-F and gTS-R, a 1676-bp fragment amplified with primers LHC-F and LHC-R, and a 1936-bp fragment amplified with primers RHC-F and RHC-R (Figure 2C). The second and third instar larvae of the F_1_ generation were screened for green fluorescence (EGFP^+^) (Figure 3A) and abnormal yellow body and head pigmentation (Y^−^) (Figure 3B). One EGFP-positive female and one EGFP-positive male with yellow mutant phenotype were subsequently obtained from 28200 F_1_ individuals and named founder 32 and founder 40, respectively (Supplementary Table S3).

Furthermore, we scored three kinds of phenotypes with corresponding genotypes such as strong EGFP fluorescence and yellowish body (EGFP^++^Y^−-^), patchy EGFP fluorescence and yellowish body (EGFP^+−^Y^--^), and patchy EGFP fluorescence and wild-type body color (EGFP^+−^Y^-+^) due to non-mendelian segregation after crossing of homozygous EGFP^++^Y^−-^individuals with wild type (EGFP^−-^Y^++^) in different generations of both founders (Figure 3). According to the Mendelian segregation, all F_1_ progeny should be heterozygote with EGFP-positive and wild-type body color because *yellow* gene is recessive. The conversion of heterozygote to homozygote progeny indicates the homing of Cas9-EGFP-gRNA component to neighboring allele.

**FIGURE 4:**
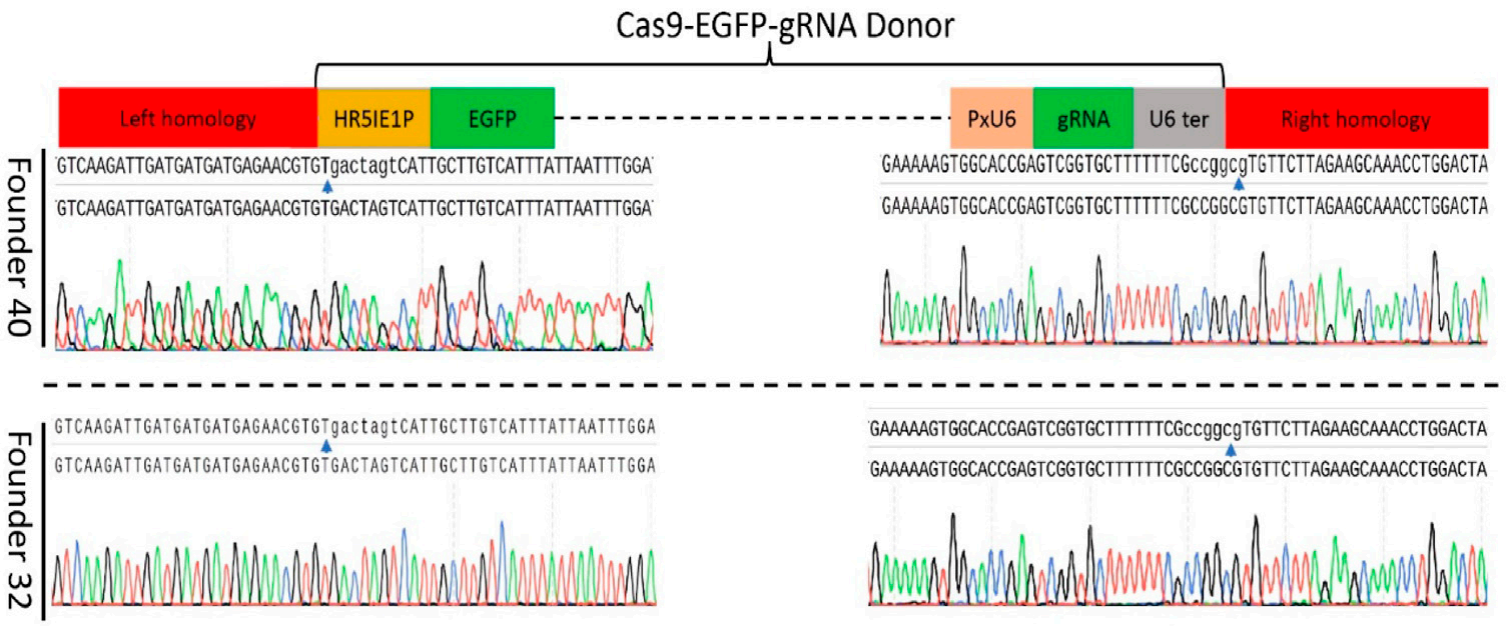
Sequence confirmation of the precise insertion of the gene-drive construct into the *Pxyellow* locus. The PCR amplification and genomic DNA sequencing were performed to verify the precise insertion of gene-drive components at *Pxyellow* locus in two F_1_ founders (40 and 32). Sequence of 5′-end fragment amplified by LH-F and LH-R is presented at the left side, and sequence of 3′-end fragment amplified by RH-F and RH-R is presented at the right side in both founders. The blue arrows represent the junction between the *Pxyellow* coding sequence and gene-drive construct.

The target-specific insertion of the gene-drive cassette at *Pxyellow* locus was confirmed in both F_1_ founders through PCR amplification (Figure 2C). The sequencing of both diagnostic amplicons containing the left and right homologous regions and their adjacent sequences in the gene-drive cassette showed cassette insertion at the *Pxyellow* target site (Figure 4 and Supplementary Appendix Table S3).

### Gene-Drive Efficiency in Different Generations of Transgenic *P. xylostella*

**FIGURE 5:**
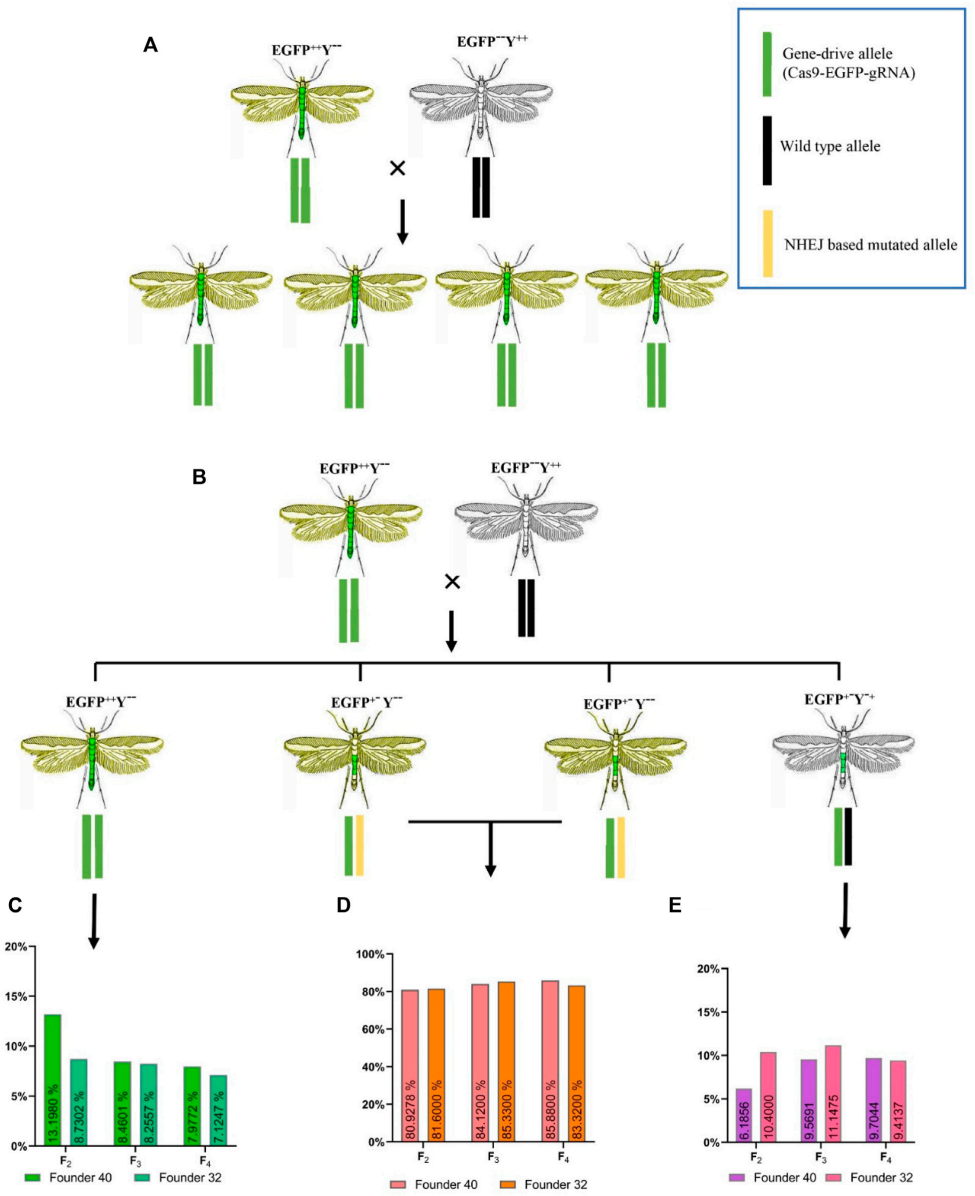
Expected and obtained phenotypes with different genotypes. **(A)**, expected genotypes and phenotypes for an ideal homing endonuclease-based gene-drive system; **(B)**, obtained genotypes and phenotypes during this study; **(C)**, the homing efficiency in different generations; **(D)**, the resistant allele formation with one allele inherited and the second allele mutated without homing; **(E)**, the resistant-allele formation with one allele inherited and the second allele uncut.

Under ideal homing endonuclease-based gene-drive conditions (100%), all progenies should show stable EGFP and yellowish phenotype (Figure 5A). Based on the above-mentioned genotypes, there were 13.19% individuals of EGFP^++^Y^−-^in F_2_ generation, 9.40% in F_3_ generation and 8.32% in F_4_ generation for founder 40. Similarly, for founder 32, there were only 8.25% individuals of EGFP^++^Y^−-^genotype in F_2_ generation, 8.91% in F_3_ generation and 7.82% in F_4_ (Figure 5B). These individuals of genotype EGFP^++^Y^−-^indicated the homing (gene-drive) of one inherited allele to neighboring wild-type allele through the HDR process. However, more than 85% of alleles were either mutated through the NHEJ process (Figure 5C) or uncut in F_2_, F_3_ and F_4_ generations from both founders (Figure 5D).

There was no difference in the homing efficiency among the different generations of both founders (founder 32, *χ* ^2^ = 2.872, *df* = 2, *p* = 0.237; founder 40, *χ* ^2^ = 0.232, *df* = 2, *p* = 0.891) or between the two founders (*χ* ^2^ = 0.073, *df* = 1, *p* = 0.7870). These results indicated that all progeny derived from parent crosses contained the drive allele, from which only 7.82%–13.19% were converted into homozygous drive allele (EGFP^++^Y^−-^) due to HDR and the rest were converted into resistance allele (EGFP^+−^Y^--,^ EGFP^+−^Y^-+^) due to NHEJ (Figure 5 C and D). This situation indicated that cleavage of the target site with Cas9 happened in somatic tissues rather than in the germline tissues, which caused the formation of resistance allele.

**FIGURE 6:**
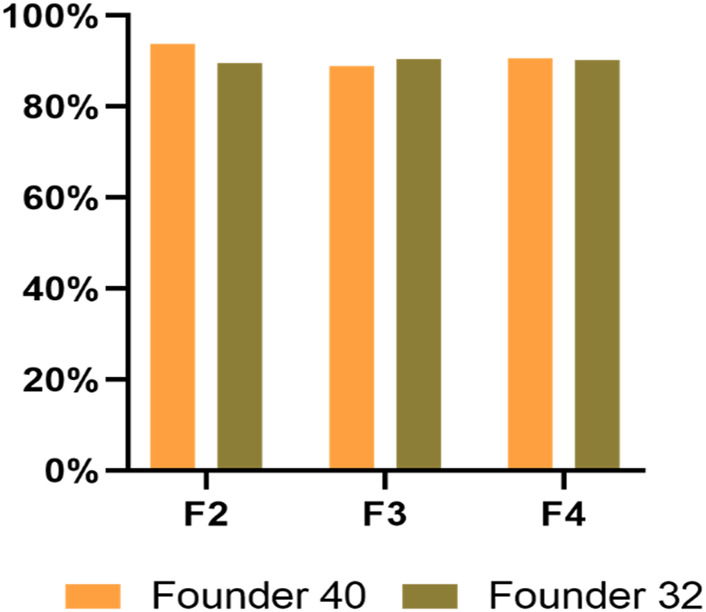
The cleavage efficiency of *Pxyellow* target site in different generations from both founders.

There was no difference in the Cas9/sgRNA cleavage efficiency of negibouring alleles between two founders (*χ* ^2^ = 1.869, *df* = 1, *p* = 0.1715) or among different generations of both founders (founder 32, *χ* ^2^ = 2.864, *df* = 2, *p* = 0.239; founder 40, *χ* ^2^ = 3.473, *df* = 2, *p* = 0.176), which all showed the cleavage efficiency of above 88% in *P. xylostella* (Figure 6).

Furthermore, the individuals with heterozygous EGFP^+−^Y^--^phenotype were randomly selected for genotype confirmation through PCR amplification. The PCR results showed the deletion of base pairs at the *Pxyellow* target site in heterozygote EGFP^+−^Y^--^mutants from both founders (Supplementary Figure S6). After cutting of genomic DNA by Cas9, EGFP^+−^Y^--^caused by NHEJ happened more frequently than EGFP^++^Y^−-^caused by HDR in F_2_ progeny (*Z* = -3.211, *p* = 0.001), in F_3_ progeny (*Z* = -2.521, *p* = 0.012) and in F_4_ progeny (*Z* = -2.521, *p* = 0.012).

### Effects of Parents on Gene-Drive Efficiency in Progeny

The cross between founder 40 (F_1_ EGFP^++^Y^−-^male) and wild-type female produced less progeny (125 adult/cross) than the cross between founder 32 (F_1_ EGFP^++^Y^−-^female) and wild-type male (194 adult/cross) (*χ* ^2^ = 7.402, *df* = 1, *p* = 0.006) (Table 1).

**TABLE 1:** Genotypes of F_2_ progeny of crosses between F_1_ EGFP^++^Y^−-^and wild type.

Cross			Genotype of progeny					
Founder (F_1_ EGFP^++^Y^−-)^	WT	Number	Female			Male		
			EGFP^++^Y^−-^	EGFP^+−^Y^--^	EGFP^+−^Y^-+^	EGFP^++^Y^−-^	EGFP^+−^Y^--^	EGFP^+−^Y^-+^
40 (**♂**)	♀	1	7	84	7	18	73	5
32 (♀)	♂	1	3	67	6	7	35	7
Total			10	151	13	25	108	12

For founder 40, crosses between F_2_ EGFP^++^Y^−-^female and wild-type male produced less progeny (147.1 adult/cross) than crosses between F_2_ EGFP^++^Y^−-^male and wild-type female (240.28 adult/cross) (*χ* ^2^ = 46.794, *df* = 1, *p* = 0.000); for founder 32, crosses between F_2_ EGFP^++^Y^−-^female and wild-type male (196.6 adult/cross) produced less progeny than crosses between F_2_ EGFP^++^Y^−-^female and wild-type male (215 adult/cross) (*χ* ^2^ = 46.581, *df* = 1, *p* = 0.000) (Table 2.

**TABLE 2:** Genotypes of F_3_ progeny of crosses between F_2_ EGFP^++^Y^−^ and wild type.

Cross				Genotype of progeny					
EGFP^++^Y^−-^parent		Number	Female				Male		
Founder	Sex		EGFP^++^Y^−-^		EGFP^+−^Y^--^	EGFP^+−^Y^-+^	EGFP^++^Y^−-^	EGFP^+−^Y^--^	EGFP^+−^Y^-+^
40	**♀**	7	14		472	78	30	401	35
	**♂**	7	64		765	87	121	592	53
	Total		78		1237	165	151	993	88
32	**♀**	3	6		291	50	21	190	32
	**♂**	4	26		412	57	79	264	22
	Total		32		703	107	100	454	54

For founder 40, crosses between F_3_ EGFP^++^Y^−-^female and wild-type male produced less progeny (188.16 adult/cross) than crosses between F_3_ EGFP^++^Y^−-^male and wild-type female (257.4 adult/cross) (*χ* ^2^ = 47.342, *df* = 1, *p* = 0.000); for founder 32, crosses between F_3_ EGFP^++^Y^−-^female and wild-type male produced less progeny (182.5 adult/cross) than crosses between F_3_ EGFP^++^Y^−-^and wild-type female (264.7 adult/cross) (*χ* ^2^ = 519.35, *df* = 1, *p* = 0.000) (Table 3).

**TABLE 3:** Genotypes of F_4_ progeny of crosses between F_3_ EGFP^++^Y^−-^and wild type.

Cross			Genotype of progeny					
EGFP^++^Y^−-^ parent		Number	Female			Male		
Founder	Sex		EGFP^++^Y^−-^	EGFP^+−^Y^−−^	EGFP^+−^Y^-+^	EGFP^++^Y^−-^	EGFP^+−^Y^--^	EGFP^+−^Y^-+^
40	**♀**	6	2	672	66	30	313	46
	**♂**	10	5	1575	63	210	678	43
	Total		7	2247	129	240	991	89
32	**♀**	6	0	548	53	34	419	41
	**♂**	10	12	1430	70	223	850	62
	Total		12	1978	123	257	1269	103

**FIGURE 7:**
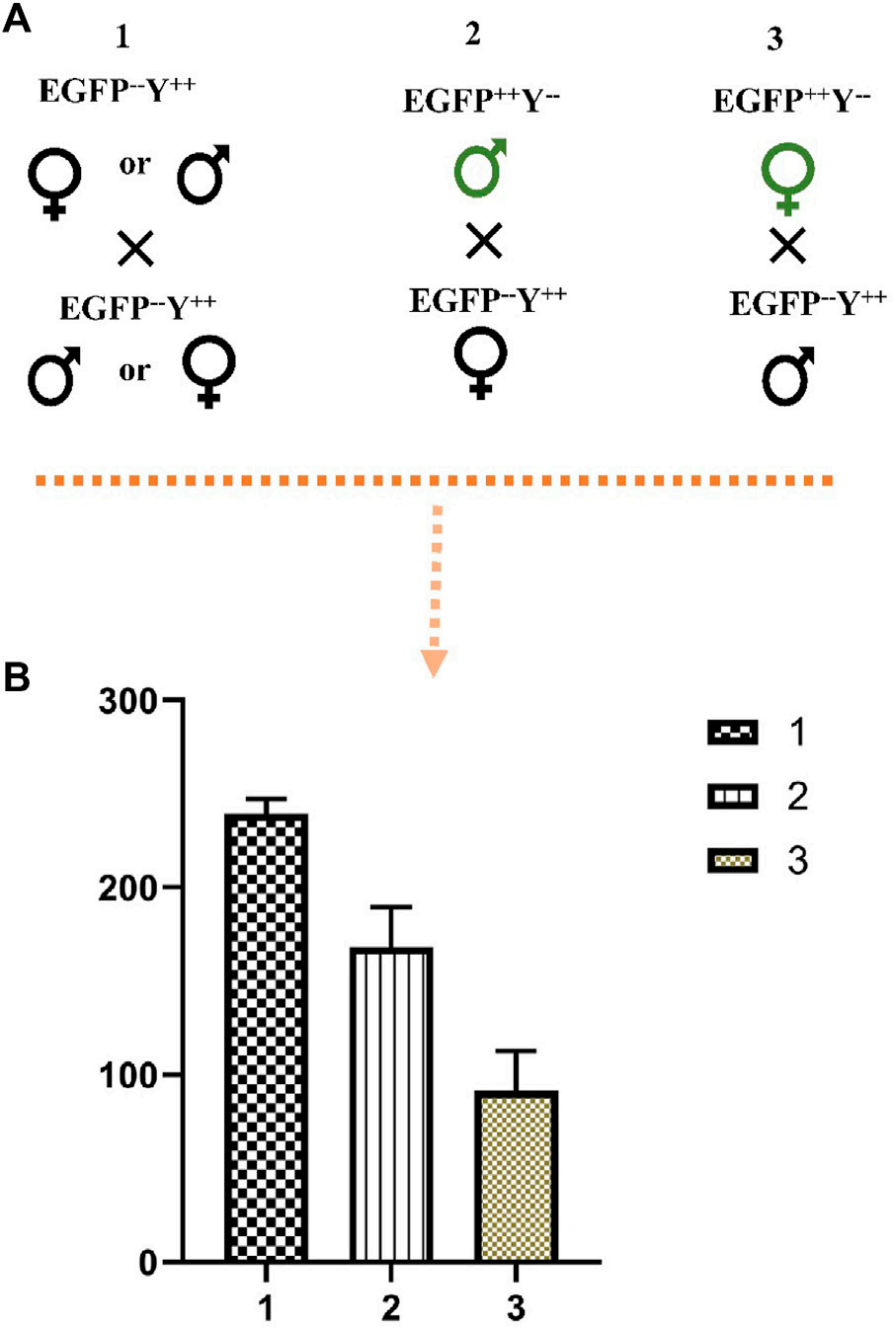
The effect of EGFP^++^Y^−-^parents on the number of progenies. **(A)**, schematic representation of crosses, **(B)**, the number of progenies produced from crosses of different parents; 1, cross between wild-type male and female; 2, cross between EGFP^++^Y^−-^male and wild-type female; 3, cross between EGFP^++^Y^−-^female and wild-type male.

The cross between wild-type male and female produced more progeny than the cross between EGFP^++^Y^−-^male and wild-type female (*χ* ^2^ = 7.33, *df* = 1, *p* = 0.003); the cross between EGFP^+^Y^−^ male produced more progeny than the cross between EGFP^++^Y^−-^female and wild-type male (*χ* ^2^ = 16.34, *df* = 1, *p* = 0.000) (Figure 7). Therefore, the above results indicated that knock-in of gene-drive cassette reduced the fecundity of females.

**FIGURE 8:**
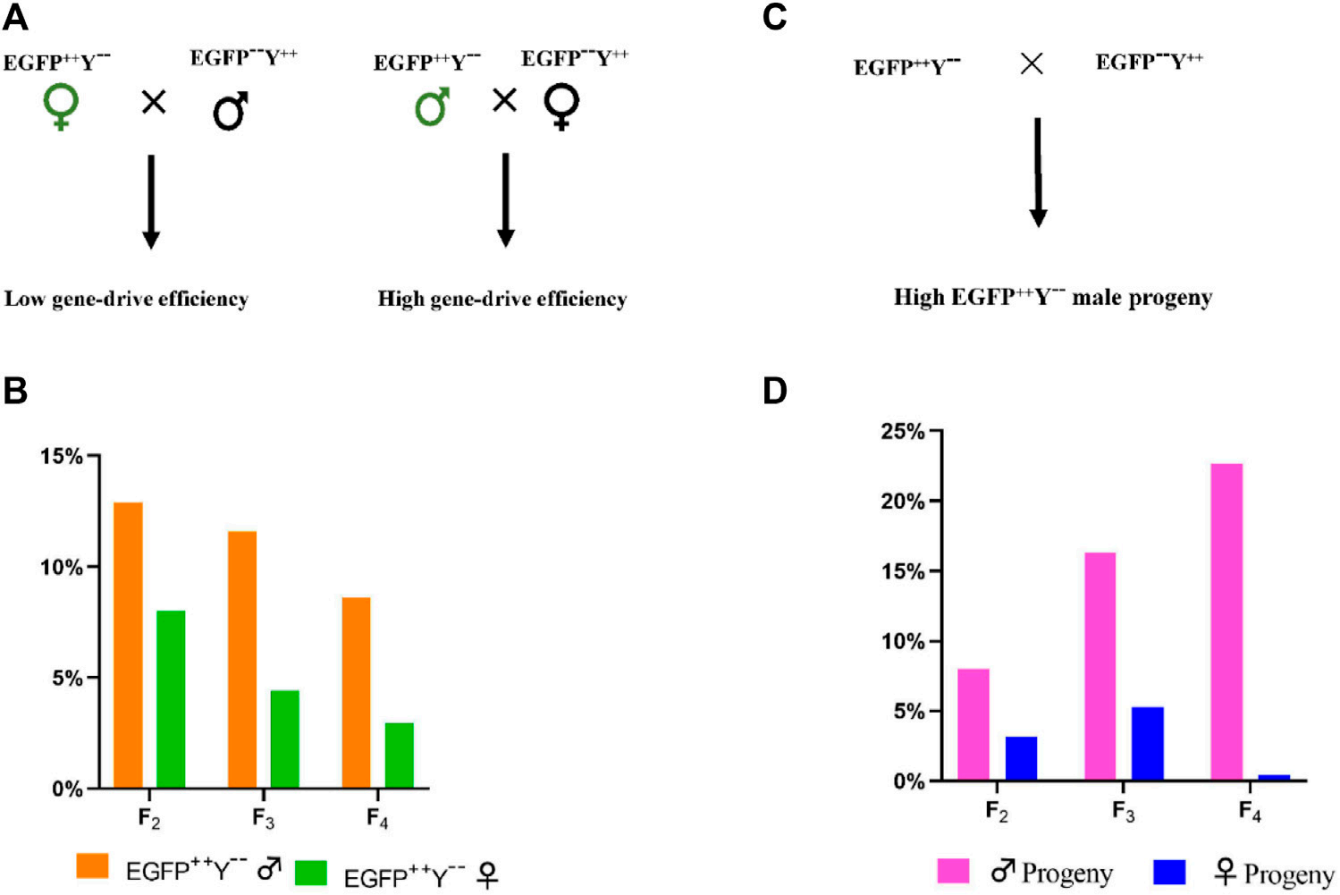
The difference in gene-drive efficiency caused by the male and female parents. **(A)**, schematic representation of EGFP^++^Y^−-^parents cross with wild-type parents, **(B)**, the gene-drive percentage caused by EGFP^++^Y^−-^male and female parents; **(C)**, representation of crosses, **(D)**, percentage of male and female progeny with EGFP^++^Y^−-^genotype.

Crosses between EGFP^++^Y^−-^female and wild-type male produced less EGFP^++^Y^−-^progeny than crosses between EGFP^++^Y^−-^male and wild-type female (F_2_, *χ* ^2^ = 7.994, *df* = 1, *p* = 0.004; F_3_, *χ* ^2^ = 62.684, *df* = 1, *p* = 0.000; F_4_, *χ* ^2^ = 104.791, *df* = 1, *p* = 0.000) (Figure 8A). Crosses between EGFP^++^Y^−-^and wild type produced less EGFP^++^Y^−-^females than EGFP^++^Y^−-^males (F_2_, *χ* ^2^ = 7.641, *df* = 1, *p* = 0.005; F_3_, *χ* ^2^ = 112.035, *df* = 1, *p* = 0.000; F_4_, *χ* ^2^ = 939.138, *df* = 1, *p* = 0.000) (Figure 8B). Therefore, the gene-drive efficiency caused by parent males was much higher than that caused by parent females. The gene-drive efficiency was much higher in male progeny than in female progeny in *P. xylostella*.

## Discussion

The *Pxyellow* gene was selected as the target site for the confirmation of phenotypic changes in *P. xylostella*. The CRISPR/Cas9-mediated knockout of *Pxyellow* gene demonstrated the change of body and head capsule color from black to yellowish. The change in body and head capsule color was quite apparent in the second and third instar larvae. A similar kind of results are previously reported, in which the *Pxyellow* gene plays an essential role in body pigmentations in *P. xylostella* (Wang et al., 2020). To develop an effective gene-drive system to evaluate drive efficiency and fitness cost, it is an appropriate way to target endogenous phenotypic marker genes, which help in screening mutant progeny. A CCGD system has previously been developed in *Drosophila melanogaster*, in which phenotypic marker *yellow* and *white* genes are used as the target sites (Champer et al., 2017; Champer et al., 2018; Champer et al., 2019). Similarly, this system has been developed in mosquitos (*Anopheles* and *Aedes*), in which the *white-eye* phenotypic marker gene is used as the target site (Gantz et al., 2015; Li et al., 2020). The selection of the phenotypic marker gene provides an easy way to screen the mutant progeny.

The gene-drive cassette worked in lepidopteran pest, *P. xylostella*. The approximately 12-kb long construct could effectively create site-specific mutation. These results are consistent with the previous study conducted in *An. gambiae*, in which approximately 17-kb construct shows its feasibility to create site-specific cleavage (Gantz et al., 2015). Additionally, the overall cleavage efficiency of neighboring allele of 88.86–93.82%, gene-drive of 6.67%–12.59%, and resistant-allele formation of 80.93%–86.77% were observed. Surprisingly, the gene-drive efficiency was much lower than other insects. Certain factors might be involved in the low gene-drive efficiency, either gene-drive component or the target site, associated with the fitness cost. The differences in the gene-drive efficiency depend upon numerous factors, such as species-specific factors, variations in the *Cas9* expression, and the genomic target site. The species-specific factor might contribute to the low gene-drive efficiency in *P. xylostella* because the enormous variations across the species were observed, such as close to 100% in yeast *Saccharomyces cerevisiae* (Dicarlo et al., 2015; Roggenkamp et al., 2018; Shapiro et al., 2018), 19%–62% in *D. melanogaster* (Champer et al., 2017; Champer et al., 2018; Karaminejadranjbar et al., 2018; Champer et al., 2019) and 87%–99% in *An. gambiae* (Gantz et al., 2015; Kyrou et al., 2018; Li et al., 2020). Therefore, different species may have different gene-drive efficiencies.

The expression of Cas9 in somatic and germline tissues also plays a crucial role in gene-drive efficiency. In *D. melanogaster*, the germline-specific *nanos* and *vasa* promoters were used to drive *Cas9* gene expression for the development of the CCGD system. The *nanos* promoter construct to drive *Cas9* exhibits a high gene-drive efficiency and a low resistant-allele formation due to the low *Cas9* expression in somatic tissues. In contrast, the construct containing *vasa* promoter to drive *Cas9* exhibits a high resistant-allele formation and a low gene-drive efficiency due to the high *Cas9* expression in somatic tissues (Champer et al., 2018). In *An. stephensi,* the *vasa* promoter used to drive the Cas9 expressions results in a high germline gene conversion through HDR-mediated repair (99%) (Gantz et al., 2015). Recently, the different germline-specific promoters of *P. xylostella* have been tested in the CRISPR/Cas9-mediated split-drive system. All these tested promoters show a high somatic cleavage with limited germline cleavage. However, no homing is observed in this CRISPR/Cas9 split drive (Xu et al., 2022). Therefore, the selection of germline-specific promoters to drive the *Cas9* expression is critical. In our study, the PxnanosP showed a high frequency of resistant-allele formation rather than gene-drive in *P. xylostella*, which might be due to a high *Cas9* expression driven by PxnanosP in somatic tissues.

Another factor that may contribute to the gene-drive efficiency is the genomic target site of gRNA. In *D. melanogaster*, the *nanos* promoter-based gene-drive construct targeting the two different genomic target sites, such as the *white* gene and *cinnabar* gene, exhibits different gene-drive efficiency (59% and 38%) (Champer et al., 2018; Champer et al., 2019). Similarly, the *vasa* promoter-based gene-drive construct targeting the two different genomic target sites, such as *yellow* gene and *yellow* gene promoter, showed 55% and 37% gene-drive efficiency, respectively (Champer et al., 2017). Hence, the different genomic target sites cause different gene-drive efficiencies. In our study, the *yellow* gene was targeted, which might contribute to a low gene-drive efficiency. Therefore, more targets should be identified and tested to achieve a high gene-drive efficiency.

The gene-drive efficiency caused by the parent male was much higher than that caused by parent female, and the gene-drive efficiency was much higher in male progeny than in female progeny in *P. xylostella*. Previous studies exhibit that the maternally-deposited *Cas9* contributes to the development of resistant alleles in *An. stephensi* (Gantz et al., 2015). Similarly, in *D. melanogaster,* resistant-allele formation is due to the high maternal-deposition of *Cas9* in eggs before fertilization or embryo development (Champer et al., 2017; Champer et al., 2018; Champer et al., 2019; Kandul et al., 2020). Therefore, the low gene-drive efficiency and high resistant-allele formation in progeny derived from transgenic females are due to the high level of maternally-deposited Cas9. The knock-in of gene-drive construct reduced the fecundity of transgenic females. The high Cas9 expression may have some side effects, reducing fecundity. Further studies are required to answer this question.

Toward the success of CRISPR/Cas9-mediated gene drive for pest control, it is essential to increase the gene-drive efficiency. The gene-drive efficiency is much lower in *P. xylostella* than in other insects. The problem of low gene-drive efficiency can be tackled with different approaches. The selection of suitable germline promoters can effectively drive Cas9 expression in germ cells and cause DSB followed by homing during gametogenesis (Hammond et al., 2016). Multiplex gRNAs targeting the different locus of a gene may increase the gene-drive efficiency (Champer et al., 2018). Suppression of NHEJ pathway genes may increase the rate of HDR (Zhu et al., 2015). As in *B. mori*, it has been described that the suppression of NHEJ-pathway genes, such as *BmKu70* and *BmKu80*, increases the HDR-mediated repair (Zhu et al., 2015). Since the gene-drive efficiency is low, further studies are required to understand the mechanism of resistant-allele formation for increasing the gene-drive efficiency.

## Conclusion

In general, our data demonstrated that the CRISPR/Cas9-mediated gene-drive construct worked in *P. xylostella* through effectively copying the gene-drive components to the target site, and the NHEJ event happened more than the HDR. The progeny derived from mutant male parents showed a relatively-high gene-drive efficiency than those from mutant female parents. These results provided a foundation for further development of the CCGD system in *P. xylostella,* especially for the pest management program. Our results also provide a valuable information for future construction of highly improved and optimized CRISPR/Cas9-mediated gene-drive for genetic control of globally-distributed pest *P. xylostella*.

## Data Availability

The original contributions presented in the study are included in the article/Supplementary Material, further inquiries can be directed to the corresponding author.
