# Supplementary material for: Development of CRISPR/Cas9-Mediated Gene-Drive Construct Targeting the Phenotypic Gene in Plutella xylostella
Source: Front Physiol. 2022 Jun 29;13:938621. doi: 10.3389/fphys.2022.938621 (PMC9277308; doi:10.3389/fphys.2022.938621)
Supplement: Supplementary file 1 [file DataSheet1.zip › Supplementary files/Supplementary Appendix.docx]

**Supplementary Appendix**

| Table 1 Genotype information of indel mutations at *Pxyellow* target site | | | |
| --- | --- | --- | --- |
| Sequence | Indel | individuals | Percentage |
| GAACGTGTGGGTTTTGTCGGACAGAATGCCGGTGTTCTTGGA | WT | - | - |
| GAACGTGTGGGTTTTGTCGGA--GAATGCCGGTGTTCTTGGA | -2bp | 11 | 18.33 |
| GAACGTGTGGGTTTTGTCGGA---AATGCCGGTGTTCTTGGA | -3bp | 5 | 8.33 |
| GAACGTGTGGGTTTTGTCGGA----ATGCCGGTGTTCTTGGA | -4bp | 8 | 13.33 |
| GAACGTGTGGGTTTTGTCGGA---------GGTGTTCTTGG | -9bp | 9 | 15.00 |
| GAACGTGTGGGTTTTGTCGGACTAGTTCAAAACCGGTGTTCTTGGA | -7bp, +11bp | 4 | 6.67 |
| GAACGTGTGGGTTTTGTCCACTATTAAACGGTACAGAATGCCGGTGTTCTTGGA | -11bp, +23bp | 6 | 10.00 |

| Table 2 Sequence detail of all components used in CRISPR/Cas9 gene-drive construct. |
| --- |
| >Left homology arm  TGAACTACATCCCTCTCGATGCTCCGTACGAGCCATCCCCCAAGTTGATCCCCTACCCCAGCTGGGAGGGCAATGAACTCGGCAACTGTCAGAATGGACTGAACACCGTCTACAGAATCAAAGCCGACAAATGTGACCGTCTCTGGGTGTTGGACGTTGGCACTTATGGTTATGGTTAGTATCAATTCTAAATAATTTACTTTCATAGTTTATAAATGATTTCCATGCCGAAATGTTTTCAATTTTACTGTTTGTAATACGACGATAGAATGACGCAGCGAATCGAGCAATATCACATCTGCCTGGTAAATTCGTAGATTAGCCACTTTCATTATTTACAAGGCTAATTGTTTTTTAATAAAACAAATGTTTTGTTTGTTTCAGATCCCAATGTTACTAACCCCTGTCCTTACGCCCTTAACGTCTATGATCTTAAAACTGACCGTCGTCTCCGGAGATACGTCTTCCGCCCTGAAGACATCGTCCCCACCACTTTCATCGCTAACATTGCTCTCGATGAAGGCAAAAGTTGCGAAGACACTTTCGCATACTTTTCCGACGAACTTGGATATGGACTCATCGCCTACTCCTGGGAGCAGAACAAATCTTGGCGGTTCACTCACAACTTCTTCATGCCTGATCCTCTTGTTGGTGAATTCAACATCGCCGGTCTGACCTTCGACTGGGGAGTGGAGGGCATATTCGGAATCTCCGCGTCGCCAGCAGGCAGCGATGGCTTCCGCACTCTCTACTTCAGTCCGCTCACCAGCCACACGGAGTTCGCCGTCTCCACCAGCGTCTTGAGAAACGAAACCAAGACCAAAGGAAACTACAAAGACTTCAGAGTAGTTGGCAACCGCGGCCCTGACACTCACACTACTGCCAAGGTCGTCGGAGAGAACGGAGTCCAACTGTACAGCTTAATCGACCAGAACGGCATTGGCTGCTGGAATATAAACAAACCCCTGAAACCGGAAAACACTGCTGTAGTTGACAAGGATGACGTTGGCTTGGTCTTCCCCAGTGATGTCAAGATTGATGATGATGAGAACGTGTG |
| >HR5IE1 promoter  CATTGCTTGTCATTTATTAATTTGGATGATGTCATTTGTTTTTAAAATTGAACTGGCTTTACGAGTAGAATTCTACGCGTAAAACACAATCAAGTATGAGTCATAAGCTGATGTCATGTTTTGCACACGGCTCATAACCGAACTGGCTTTACGAGTAGAATTCTACTTGTAACGCACGATCAGTGGATGATGTCATTTGTTTTTCAAATCGAGATGATGTCATGTTTTGCACACGGCTCATAAACTCGCTTTACGAGTAGAATTCTACGTGTAACGCACGATCGATTGATGAGTCATTTGTTTTGCAATATGATATCATACAATATGACTCATTTGTTTTTCAAAACCGAACTTGATTTACGGGTAGAATTCTACTTGTAAAGCACAATCAAAAAGATGATGTCATTTGTTTTTCAAAACTGAACTCGCTTTACGAGTAGAATTCTACGTGTAAAACACAATCAAGAAATGATGTCATTTGTTATAAAAATAAAAGCTGATGTCATGTTTTGCACATGGCTCATAACTAAACTCGCTTTACGGGTAGAATTCTACGCGTAAAACATGATTGATAATTAAATAATTCATTTGCAAGCTATACGTTAAATCAAACGGACGCTCGAGGTTGCACAACACTATTATCGATTTGCAGTTCGGGACATAAATGTTTAAATATATCGATGTCTTTGTGATGCGCGCGACATTTTTGTAGGTTATTGATAAAATGAACGGATACGTTGCCCGACATTATCATTAAATCCTTGGCGTAGAATTTGTCGGGTCCATTGTCCGTGTGCGCTAGTAGCATGCCCGTAACGGACCTCGTACTTTTGGCTTCAAAGGTTTTGCGCACAGACAAAATGTGCCACACTTGCAGCTCTGCATGTGTGCGCGTTACCACAAATCCCAACGGCGCAGTGTACTTGTTGTATGCAAATAAATCTCGATAAAGGCGCGGCGCGCGAATGCAGCTGATCACGTACGCTCCTCGTGTTCCGTTCAAGGACGGTGTTACCGACCTCAGATTAATGTTTATCGGCCGACTGTTTTCGTATCCGCTCACCAAACGCGTTTTTGCATTAACATTGTATGTCGGCGGATGTTCTATATCTAATTTGAATAAATAAACGATAACCGCGTTGGTTTTAGAGGGCATAATAAAAGAAATATTGTTATCGTGTTCGCCATTAGGGCAGTATAAATTGACGTTCATGTTGGATATTGTTTCAGTTGCAAGTTGACACTGGCGGCGACAAGA |
| >EGFP  ATGGTGAGCAAGGGCGAGGAGCTGTTCACCGGGGTGGTGCCCATCCTGGTCGAGCTGGACGGCGACGTAAACGGCCACAAGTTCAGCGTGTCCGGCGAGGGCGAGGGCGATGCCACCTACGGCAAGCTGACCCTGAAGTTCATCTGCACCACCGGCAAGCTGCCCGTGCCCTGGCCCACCCTCGTGACCACCCTGACCTACGGCGTGCAGTGCTTCAGCCGCTACCCCGACCACATGAAGCAGCACGACTTCTTCAAGTCCGCCATGCCCGAAGGCTACGTCCAGGAGCGCACCATCTTCTTCAAGGACGACGGCAACTACAAGACCCGCGCCGAGGTGAAGTTCGAGGGCGACACCCTGGTGAACCGCATCGAGCTGAAGGGCATCGACTTCAAGGAGGACGGCAACATCCTGGGGCACAAGCTGGAGTACAACTACAACAGCCACAACGTCTATATCATGGCCGACAAGCAGAAGAACGGCATCAAGGTGAACTTCAAGATCCGCCACAACATCGAGGACGGCAGCGTGCAGCTCGCCGACCACTACCAGCAGAACACCCCCATCGGCGACGGCCCCGTGCTGCTGCCCGACAACCACTACCTGAGCACCCAGTCCGCCCTGAGCAAAGACCCCAACGAGAAGCGCGATCACATGGTCCTGCTGGAGTTCGTGACCGCCGCCGGGATCACTCTCGGCATGGACGAGCTGTACAAG |
| >Pxnanos promoter  TAGTGACGTCTCCTGGAGTGAGGTAACACTTGATTCTTCCTCGTGCGGCACACCGCGGCCAGTCAAAGACTGATGCTAGTCTCAATGGCCAATCCTTAGGCCGCGGTGACAAACCCTATTCTACTACCTACTACCTAGTCAGGCTTTACTCTAGTTGTACTTTGGATTTTTAAAAGCTGTAAGGCTTATGATACCTAAACACAGCTGGAGATAATAATATGTCCGTTTTCCATACTGATAATGGAAATTACTTTCATTTAAAACTATGACAATGGTCTAAGCAAATAAAAATAAATGCTTGACCTTACAAGAACAGATTTTGAAATTTCGTTTAGTGCCACCTAGTGCTATTTAGTTGAACTATGTAAGTAGCAAACCACACTACGGAAGTAAATTACGTGCATTGGCATATTTGGCACAAAGTAGATGAATAGGAAACTAAGTAATTTAACGTAAGCCCGATAGATGGCAGTAAATACATTTTCAGGTTGGCATGGTAAATTATTTCAACTCATTCATTATTGTCAGATTTTTTAACAATTTCTGCAAACCAACAAACGAATTACATGTATTTTTGGAATAAAGGGATTTTTGTCACCTTTATTTGAAACCATTTTCACACAACTATACAATACCTATTTGCCTTATCCGTCCTAGGTTGCCCTAGGTAGGTTAGGTAAGTACCTACTTAGTTATTTAGTTAGGTACTTACACCTCGATCGTTTTTTTACACTGAGACTGGTAAGTTGGTAACCGACTGTTTCAAATTATTTTATGAAACTTTTTTAACCTTTCAATAACTACCTAGGTAAGTACTTACCAACTTACTTGATTTCGTGGACCTCAAAACTCCATTCAACTCATTCATCACAACGAGTTTTAAGTATTCCTCAAGTAGTTAAAAACTCAAGCACTTAGAGGCCCTCACAAAGTTACTCCACCATGGTGCCAACTGCCAACTGCCACCCAACCACCCAACAATAAACAACATAATTATTATACTCGACTTCACTCGAGTTCATCGAGCATAGTTGGTCTAAGTACCTACCTATGTAGGTAGGATGTAAAAAACTAACAGTATGGAGTTAACTTAATCCTCAAACTACAATTAAATACATCTACTTACAGATACTTGTTGATTAAGTATGTAGGTAGGTACTTAGTAAGCTAACTAATATTATTCGATCAGATTACGATTTCAATTGGAACTCTTTGTTTATGCCGGCTGGCGGCGGCGGCTGGTGTGGGGCGAGCGGCATCCATGAGATTATGATACCTTACATTACCTATTGATATCCGAGTCTGAATTATTAGTGTCAAACAAAAATATCAATGCAAAAGCACCGACTCAGCATTTAATATAATAAAATTAACTAATTACCCCAGGCGACAGTGTTAGTTATTCTTATAACCTACTCATTTAATTTATTTCTAACTAACTATGTAGTACTATGTAGTCTATGTGTATGTACCCTAAGTTTAGGTTCGCTTGATTAGATTTCGTTCGTCCAAGTTGATGTTGTTTTTGTAACAACATTATTGCCCTTTCTTTGCCGCTCGGCCCGGCTCTGATTTCATTGGTCGAAATTTCAAACTAGAGATTACGCTGTCCCATCGCCGGCCAATGAGCGAAAAGCATTCTCATTTCAAATCCTGCGCAGGGTGGACGCGTTCTGCTGTTCTAAATTTTTTATTAAAACAAATCATCGATCGATTGCATTGTTTATTTTAACTTTTTACAAAGGGCCC |
| >Cas9  ATGGACAAGAAGTACTCCATTGGGCTCGATATCGGCACAAACAGCGTCGGCTGGGCCGTCATTACGGACGAGTACAAGGTGCCGAGCAAAAAATTCAAAGTTCTGGGCAATACCGATCGCCACAGCATAAAGAAGAACCTCATTGGCGCCCTCCTGTTCGACTCCGGGGAGACGGCCGAAGCCACGCGGCTCAAAAGAACAGCACGGCGCAGATATACCCGCAGAAAGAATCGGATCTGCTACCTGCAGGAGATCTTTAGTAATGAGATGGCTAAGGTGGATGACTCTTTCTTCCATAGGCTGGAGGAGTCCTTTTTGGTGGAGGAGGATAAAAAGCACGAGCGCCACCCAATCTTTGGCAATATCGTGGACGAGGTGGCGTACCATGAAAAGTACCCAACCATATATCATCTGAGGAAGAAGCTTGTAGACAGTACTGATAAGGCTGACTTGCGGTTGATCTATCTCGCGCTGGCGCATATGATCAAATTTCGGGGACACTTCCTCATCGAGGGGGACCTGAACCCAGACAACAGCGATGTCGACAAACTCTTTATCCAACTGGTTCAGACTTACAATCAGCTTTTCGAAGAGAACCCGATCAACGCATCCGGAGTTGACGCCAAAGCAATCCTGAGCGCTAGGCTGTCCAAATCCCGGCGGCTCGAAAACCTCATCGCACAGCTCCCTGGGGAGAAGAAGAACGGCCTGTTTGGTAATCTTATCGCCCTGTCACTCGGGCTGACCCCCAACTTTAAATCTAACTTCGACCTGGCCGAAGATGCCAAGCTTCAACTGAGCAAAGACACCTACGATGATGATCTCGACAATCTGCTGGCCCAGATCGGCGACCAGTACGCAGACCTTTTTTTGGCGGCAAAGAACCTGTCAGACGCCATTCTGCTGAGTGATATTCTGCGAGTGAACACGGAGATCACCAAAGCTCCGCTGAGCGCTAGTATGATCAAGCGCTATGATGAGCACCACCAAGACTTGACTTTGCTGAAGGCCCTTGTCAGACAGCAACTGCCTGAGAAGTACAAGGAAATTTTCTTCGATCAGTCTAAAAATGGCTACGCCGGATACATTGACGGCGGAGCAAGCCAGGAGGAATTTTACAAATTTATTAAGCCCATCTTGGAAAAAATGGACGGCACCGAGGAGCTGCTGGTAAAGCTTAACAGAGAAGATCTGTTGCGCAAACAGCGCACTTTCGACAATGGAAGCATCCCCCACCAGATTCACCTGGGCGAACTGCACGCTATCCTCAGGCGGCAAGAGGATTTCTACCCCTTTTTGAAAGATAACAGGGAAAAGATTGAGAAAATCCTCACATTTCGGATACCCTACTATGTAGGCCCCCTCGCCCGGGGAAATTCCAGATTCGCGTGGATGACTCGCAAATCAGAAGAGACCATCACTCCCTGGAACTTCGAGGAAGTCGTGGATAAGGGGGCCTCTGCCCAGTCCTTCATCGAAAGGATGACTAACTTTGATAAAAATCTGCCTAACGAAAAGGTGCTTCCTAAACACTCTCTGCTGTACGAGTACTTCACAGTTTATAACGAGCTCACCAAGGTCAAATACGTCACAGAAGGGATGAGAAAGCCAGCATTCCTGTCTGGAGAGCAGAAGAAAGCTATCGTGGACCTCCTCTTCAAGACGAACCGGAAAGTTACCGTGAAACAGCTCAAAGAAGACTATTTCAAAAAGATTGAATGTTTCGACTCTGTTGAAATCAGCGGAGTGGAGGATCGCTTCAACGCATCCCTGGGAACGTATCACGATCTCCTGAAAATCATTAAAGACAAGGACTTCCTGGACAATGAGGAGAACGAGGACATTCTTGAGGACATTGTCCTCACCCTTACGTTGTTTGAAGATAGGGAGATGATTGAAGAACGCTTGAAAACTTACGCTCATCTCTTCGACGACAAAGTCATGAAACAGCTCAAGAGGCGCCGATATACAGGATGGGGGCGGCTGTCAAGAAAACTGATCAATGGGATCCGAGACAAGCAGAGTGGAAAGACAATCCTGGATTTTCTTAAGTCCGATGGATTTGCCAACCGGAACTTCATGCAGTTGATCCATGATGACTCTCTCACCTTTAAGGAGGACATCCAGAAAGCACAAGTTTCTGGCCAGGGGGACAGTCTTCACGAGCACATCGCTAATCTTGCAGGTAGCCCAGCTATCAAAAAGGGAATACTGCAGACCGTTAAGGTCGTGGATGAACTCGTCAAAGTAATGGGAAGGCATAAGCCCGAGAATATCGTTATCGAGATGGCCCGAGAGAACCAAACTACCCAGAAGGGACAGAAGAACAGTAGGGAAAGGATGAAGAGGATTGAAGAGGGTATAAAAGAACTGGGGTCCCAAATCCTTAAGGAACACCCAGTTGAAAACACCCAGCTTCAGAATGAGAAGCTCTACCTGTACTACCTGCAGAACGGCAGGGACATGTACGTGGATCAGGAACTGGACATCAATCGGCTCTCCGACTACGACGTGGATCATATCGTGCCCCAGTCTTTTCTCAAAGATGATTCTATTGATAATAAAGTGTTGACAAGATCCGATAAAAATAGAGGGAAGAGTGATAACGTCCCCTCAGAAGAAGTTGTCAAGAAAATGAAAAATTATTGGCGGCAGCTGCTGAACGCCAAACTGATCACACAACGGAAGTTCGATAATCTGACTAAGGCTGAACGAGGTGGCCTGTCTGAGTTGGATAAAGCCGGCTTCATCAAAAGGCAGCTTGTTGAGACACGCCAGATCACCAAGCACGTGGCCCAAATTCTCGATTCACGCATGAACACCAAGTACGATGAAAATGACAAACTGATTCGAGAGGTGAAAGTTATTACTCTGAAGTCTAAGCTGGTCTCAGATTTCAGAAAGGACTTTCAGTTTTATAAGGTGAGAGAGATCAACAATTACCACCATGCGCATGATGCCTACCTGAATGCAGTGGTAGGCACTGCACTTATCAAAAAATATCCCAAGCTTGAATCTGAATTTGTTTACGGAGACTATAAAGTGTACGATGTTAGGAAAATGATCGCAAAGTCTGAGCAGGAAATAGGCAAGGCCACCGCTAAGTACTTCTTTTACAGCAATATTATGAATTTTTTCAAGACCGAGATTACACTGGCCAATGGAGAGATTCGGAAGCGACCACTTATCGAAACAAACGGAGAAACAGGAGAAATCGTGTGGGACAAGGGTAGGGATTTCGCGACAGTCCGGAAGGTCCTGTCCATGCCGCAGGTGAACATCGTTAAAAAGACCGAAGTACAGACCGGAGGCTTCTCCAAGGAAAGTATCCTCCCGAAAAGGAACAGCGACAAGCTGATCGCACGCAAAAAAGATTGGGACCCCAAGAAATACGGCGGATTCGATTCTCCTACAGTCGCTTACAGTGTACTGGTTGTGGCCAAAGTGGAGAAAGGGAAGTCTAAAAAACTCAAAAGCGTCAAGGAACTGCTGGGCATCACAATCATGGAGCGATCAAGCTTCGAAAAAAACCCCATCGACTTTCTCGAGGCGAAAGGATATAAAGAGGTCAAAAAAGACCTCATCATTAAGCTTCCCAAGTACTCTCTCTTTGAGCTTGAAAACGGCCGGAAACGAATGCTCGCTAGTGCGGGCGAGCTGCAGAAAGGTAACGAGCTGGCACTGCCCTCTAAATACGTTAATTTCTTGTATCTGGCCAGCCACTATGAAAAGCTCAAAGGGTCTCCCGAAGATAATGAGCAGAAGCAGCTGTTCGTGGAACAACACAAACACTACCTTGATGAGATCATCGAGCAAATAAGCGAATTCTCCAAAAGAGTGATCCTCGCCGACGCTAACCTCGATAAGGTGCTTTCTGCTTACAATAAGCACAGGGATAAGCCCATCAGGGAGCAGGCAGAAAACATTATCCACTTGTTTACTCTGACCAACTTGGGCGCGCCTGCAGCCTTCAAGTACTTCGACACCACCATAGACAGAAAGCGGTACACCTCTACAAAGGAGGTCCTGGACGCCACACTGATTCATCAGTCAATTACGGGGCTCTATGAAACAAGAATCGACCTCTCTCAGCTCGGTGGAGAC |
| >PxU6 promoter  AGGAGTCCCATACCTAACAGTAAGCAATAGGAGGCTGATGATGATGATATTGTTTCAGGTGGGCAACGGAGACCTCGGTCTGGCGGGCGGAGTGGCGCTCACCGAGGACATGCTGGTCGTGGCCGACACCAGCGTCAAGATATACGACCTGCAAGGGAACCTGAAGGCCACCATGGCGCCAGTACCCAAAGGTATTTTATGCAAATATACTTGTCTAAAGTATGGCCGTTGATAGAATGCATTTTTCACATTCGGAATTCCATAGATATTTGATGACTGCAATGGAACGAACGTCAAACACGCATTATCTCAACGGCCGAAGTATAAAAACTACTAACATTACGAGTAGTTACTGAAACACTCACTAGATGGCGTTGACTAACTGATTGTATGGACTTAAGTGCTTGTATTTGTATAGGATCCTACATCGCGGTATTAAAGTTTTTTAGATCCGTCAGTACATATAGAAATTTGAATCCCTCATTAGT |
| gRNA  GGTTTTGTCGGACAGAATGC |
| >Right homology arm  TGTTCTTAGAAGCAAACCTGGACTACAGTGACATCAACTTCAGAATATACACTGCACCTCTGAGTGTGCTACTGCAAGGAACAGTGTGCGAGCCGTCACAGGCGCTGAAGCCCTACCAGGCCCCGCAAGCCCCGGACCTGCCTGCCCTCCCCCGCTTCCCCCAACTCGGGTCACCGAACTCGCAGTTTGGTCTGGCACCAGGCTCACCAAAGCAATACGAAGTGCCTCAGTACGTAATCGTGCCATCTCAAGGTCAAAAGCCACAGACGTACCTTCCGCTAATCACTGACAACTTCGCTCCTAAAGTGGAGGCTTACATCAATGTCCCGAAGCAGCCGAGCGTGCCTTACATTACAGGACGTCTTCCCAAAGCTCAGCAAAACCTTCAGTCTATTCAGAGCAACCCTGGATCTTACAGCCAGGGGCAACCAAACGCAGCATCGCCGAACCGAAACCAAAAGCCTTGGTGGTTGAGAGGGAACTACGAGGTATATGAATCATGATGAGGAAATATGTGTGTGTGTAATATTGTGTTGATGTTTGTGACAAAACGTTAGTAGTACAATAGTTATGCTATCGTGTATCTAGATGTATCTGCTGATGTTTTACGAGTATGCACGTGCGAGTGAGTGTTGACAGAGTGATTGCAAGTGCTGAATTAGGAGTGAATGATGTTTATCGCATTAGATATTGGCTTTATTAAATGCAAGCACTAGGTACCTGCTAGCTTGTTAATTAGATTAATGTATAGAAATGCGTATGAGGCTATTAATGTATTTTTTTATTTGTGCAACTTTATTCTCATTGTACCTGTTATCTTAATTCGTGTGATTGTTGTGTATGTTTCTGCACGTAATAATAACGTCGGTATGTTTACTTCTAGAATGTATTTTTTGTAAATTATATTAGAATGTATGTAGTGTTCCATTTCATAGTACTTTTATTAAATTACCATTGTATGATGAATACTCAAGCGTCCTATACTCAAGGACAAATATTGTA |

| Table 3 Sequence information of precise insertion of gene-drive construct at *Pxyellow* target site. | |
| --- | --- |
| Upstream insertion of gene-drive construct (Sequence of amplified fragment with LHC-F and LHC-R primers) | Downstream insertion of gene-drive construct (Sequence of amplified fragment with RHC-F and RHC-R primers) |
| TCTGATACACATTTGTATGCTATGATATATAGCTTATATTTACATATATACTTCAAGTAATGGTGGACCACCAAACCACCATCTAAACAGTTGAACTTTATGTTAAATTTAATCTTTATATTCTCAGGTCTAGGTTCTCCTTAACTAAAATTAATTTGAAAGTGTTGCTAAACCAGTTTTTTTATCTATTCCAGGTATCTGAACTACATCCCTCTCGATGCTCCGTACGAGCCATCCCCCAAGTTGATCCCCTACCCCAGCTGGGAGGGCAATGAACTCGGCAACTGTCAGAATGGACTGAACACCGTCTACAGAATCAAAGCCGACAAATGTGACCGTCTCTGGGTGTTGGACGTTGGCACTTATGGTTATGGTTAGTATCAATTCTAAATAATTTACTTTCATAGTTTATAAATGATTTCCATGCCGAAATGTTTTCAATTTTACTGTTTGTAATACGACGATAGAATGACGCAGCGAATCGAGCAATATCACATCTGCCTGGTAAATTCGTAGATTAGCCACTTTCATTATTTACAAGGCTAATTGTTTTTTAATAAAACAAATGTTTTGTTTGTTTCAGATCCCAATGTTACTAACCCCTGTCCTTACGCCCTTAACGTCTATGATCTTAAAACTGACCGTCGTCTCCGGAGATACGTCTTCCGCCCTGAAGACATCGTCCCCACCACTTTCATCGCTAACATTGCTCTCGATGAAGGCAAAAGTTGCGAAGACACTTTCGCATACTTTTCCGACGAACTTGGATATGGACTCATCGCCTACTCCTGGGAGCAGAACAAATCTTGGCGGTTCACTCACAACTTCTTCATGCCTGATCCTCTTGTTGGTGAATTCAACATCGCCGGTCTGACCTTCGACTGGGGAGTGGAGGGCATATTCGGAATCTCCGCGTCGCCAGCAGGCAGCGATGGCTTCCGCACTCTCTACTTCAGTCCGCTCACCAGCCACACGGAGTTCGCCGTCTCCACCAGCGTCTTGAGAAACGAAACCAAGACCAAAGGAAACTACAAAGACTTCAGAGTAGTTGGCAACCGCGGCCCTGACACTCACACTACTGCCAAGGTCGTCGGAGAGAACGGAGTCCAACTGTACAGCTTAATCGACCAGAACGGCATTGGCTGCTGGAATATAAACAAACCCCTGAAACCGGAAAACACTGCTGTAGTTGACAAGGATCGTTGGCTTGGTCTTCCCCAGTGATGTCAAGATTGATGATGATGAGAACGTGTGACTAGTCATTGCTTGTCATTTATTAATTTGGATGATGTCATTTGTTTTTAAAATTGAACTGGCTTTACGAGTAGAATTCTACGCGTAAAACACAATCAAGTATGAGTCATAAGCTGATGTCATGTTTTGCACACGGCTCATAACCGAACTGGCTTTACGAGTAGAATTCTACTTGTAACGCACGATCAGTGGATGATGTCATTTGTTTTTCAAATCGAGATGATGTCATGTTTTGCACACGGCTCATAAACTCGCTTTACGAGTAGAATTCTACGTGTAACGCACGATCGATTGATGAGTCATTTGTTTTGCAATATGATATCATACAATATGACTCATTTGTTTTTCAAAACCGAACTTGATTTACGGGTAGAATTCTACTTGTAAAGCACAATCAAAAAGATGATGTCATTTGTTTTTCA | ATACGACCTGCAAGGGAACCTGAAGGCCACCATGGCGCCAGTACCCAAAGGTATTTTATGCAAATATACTTGTCTAAAGTATGGCCGTTGATAGAATGCATTTTTCACATTCGGAATTCCATAGATATTTGATGACTGCAATGGAACGAACGTCAAACACGCATTATCTCAACGGCCGAAGTATAAAAACTACTAACATTACGAGTAGTTACTGAAACACTCACTAGATGGCGTTGACTAACTGATTGTATGGACTTAAGTGCTTGTATTTGTATAGGATCCTACATCGCGGTATTAAAGTTTTTTAGATCCGTCAGTACATATAGAAATTTGAATCCCTCATTAGTGGTTTTGTCGGACAGAATGCGTTTTAGAGCTAGAAATAGCAAGTTAAAATAAGGCTAGTCCGTTATCAACTTGAAAAAGTGGCACCGAGTCGGTGCTTTTTTCGCCGGCGTGTTCTTAGAAGCAAACCTGGACTACAGTGACATCAACTTCAGAATATACACTGCACCTCTGAGTGTGCTACTGCAAGGAACAGTGTGCGAGCCGTCACAGGCGCTGAAGCCCTACCAGGCCCCGCAAGCCCCGGACCTGCCTGCCCTCCCCCGCTTCCCCCAACTCGGGTCACCGAACTCGCAGTTTGGTCTGGCACCAGGCTCACCAAAGCAATACGAAGTGCCTCAGTACGTAATCGTGCCATCTCAAGGTCAAAAGCCACAGACGTACCTTCCGCTAATCACTGACAACTTCGCTCCTAAAGTGGAGGCTTACATCAATGTCCCGAAGCAGCCGAGCGTGCCTTACATTACAGGACGTCTTCCCAAAGCTCAGCAAAACCTTCAGTCTATTCAGAGCAACCCTGGATCTTACAGCCAGGGGCAACCAAACGCAGCATCGCCGAACCGAAACCAAAAGCCTTGGTGGTTGAGAGGGAACTACGAGGTATATGAATCATGATGAGGAAATATGTGTGTGTGTAATATTGTGTTGATGTTTGTGACAAAACGTTAGTAGTACAATAGTTATGCTATCGTGTATCTAGATGTATCTGCTGATGTTTTACGAGTATGCACGTGCGAGTGAGTGTTGACAGAGTGATTGCAAGTGCTGAATTAGGAGTGAATGATGTTTATCGCATTAGATATTGGCTTTATTAAATGCAAGCACTAGGTACCTGCTAGCTTGTTAATTAGATTAATGTATAGAAATGCGTATGAGGCTATTAATGTATTTTTTTATTTGTGCAACTTTATTCTCATTGTACCTGTTATCTTAATTCGTGTGATTGTTGTGTATGTTTCTGCACGTAATAATAACGTCGGTATGTTTACTTCTAGAATGTATTTTTTGTAAATTATATTAGAATGTATGTAGTGTTCCATTTCATAGTACTTTTATTAAATTACCATTGTATGATGAATACTCAAGCGTCCTATACTCAAGGACAAATATTGTAATAACAATTCACCATTACATGATGGAACTCTTAATATACTTTTTTACCTTTTGTACTTTACTATTTTATTGTTCTAGCGTATTATGCAAATATACATTCATTTTTACTTTTATAGGTACAATTGATGTTTTATTTCTACCGGTATACTAAGTACGGTGAAGGAAAACATCGTGAGGAAACCTGCATATCTAGACTTAGCACATCTAGATATGTGAATCCACCAACCCGCAGTGGACCAGCGTGGTGGGAAATGGTCCAAGCTTAGGAAGGCAGTTTAGACCTTGGGGATATGCACAAAGGTTCCACTCGAGAGAGCCAGGTGCAGGTACTTACACCCCCACAGAGAATAGAATAGAATAGAATACTAAGTACTTCTTCAAGGCAGAGATTGAAGGTGCTACTAATAAAATTGTTTAATATCATTCCATGATAATTATCACAATTTATAATGTGTGTTGGTTCCTCTATTTCACTCGC |
| Blue is *Pxyellow* gene sequence outside the left and right homologous arms.  Green is homologous arms sequence.  Red is HR5IE1 promoter sequence  Dark grey is PxU6 promoter and gRNA sequence. | |
