# Supplementary material for: Development of CRISPR/Cas9-Mediated Gene-Drive Construct Targeting the Phenotypic Gene in Plutella xylostella
Source: Front Physiol. 2022 Jun 29;13:938621. doi: 10.3389/fphys.2022.938621 (PMC9277308; doi:10.3389/fphys.2022.938621)
Supplement: Supplementary file 1 [file DataSheet1.zip › Supplementary files/Supplementary Figures.docx]

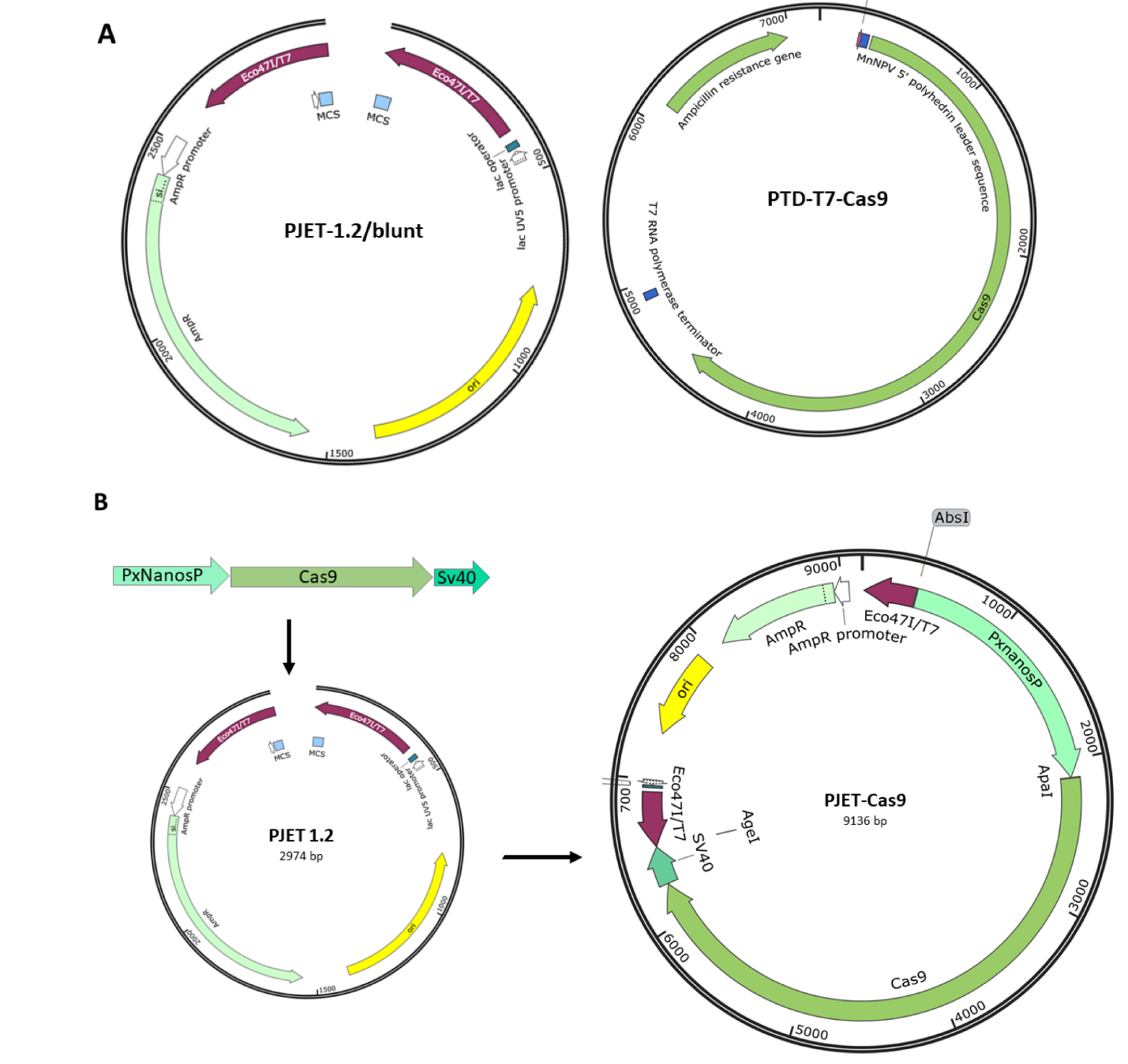


Figure S1 Schematic representation for the development of PJET-Cas9 vector by inserting the Cas9 expression cassette into the PJET 1.2 blunt-end vector. A, schematic representation of PJET-1.2 blunt-end vector and PTD-1-Cas9 expression vector; B, insertion of three fragments into PJET-1.2 blunt-end vector.


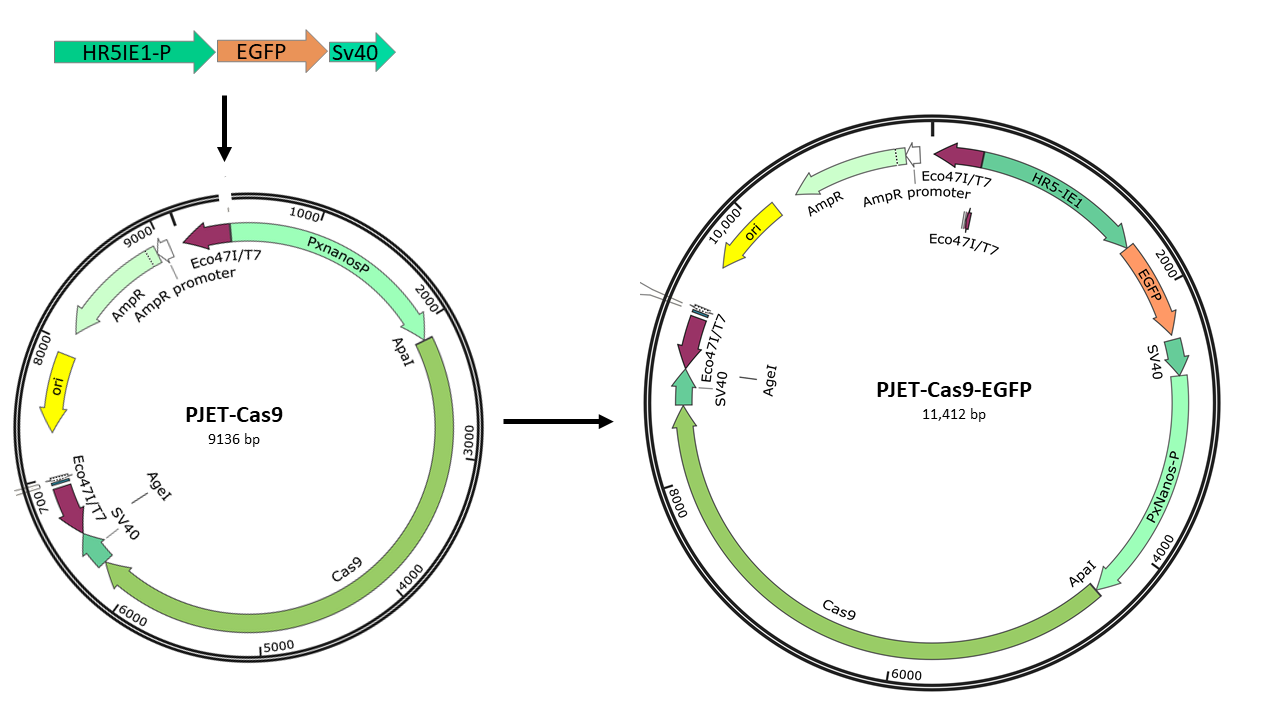


Figure S2 Schematic representation for the insertion of Hr5IE1-EGFP fragment into the PJET-Cas9 vector to obtain the PJET-Cas9-EGFP vector.


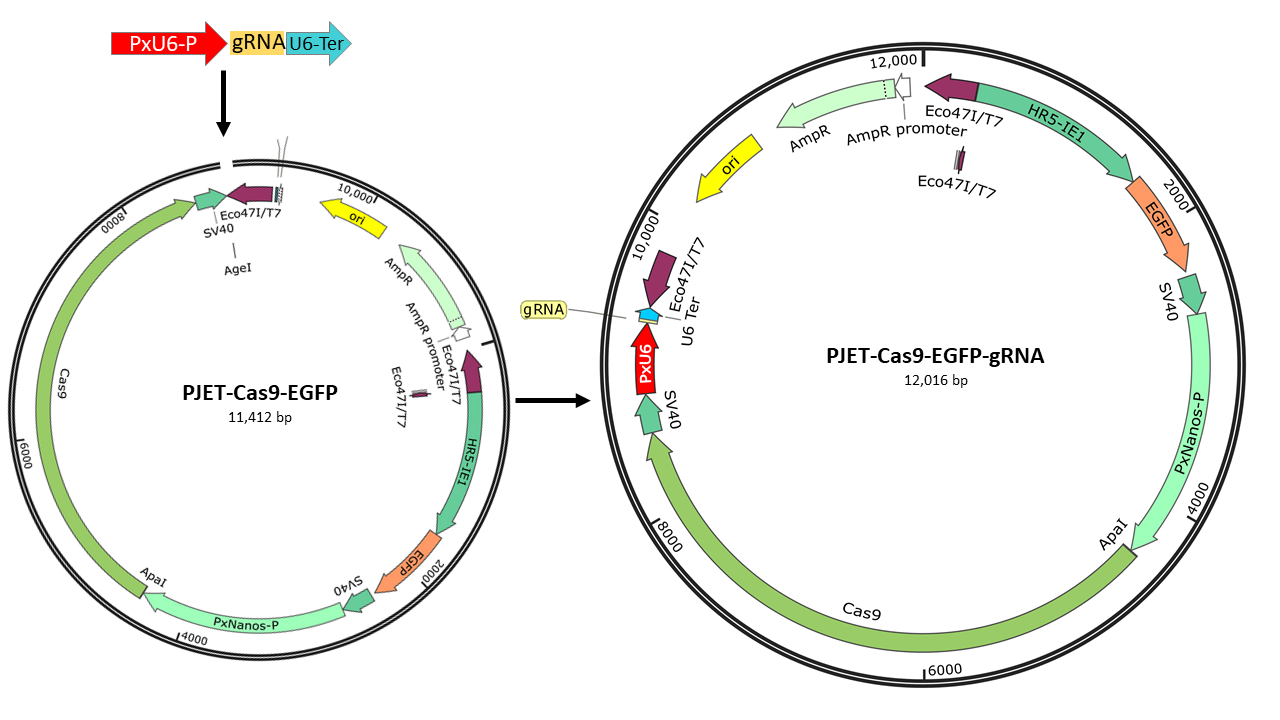


Figure S3 Schematic representation for insertion of PxU6*-*gRNA fragment to PJET-Cas9-EGFP vector to obtain the PJET-Cas9-EGFP-gRNA vector.


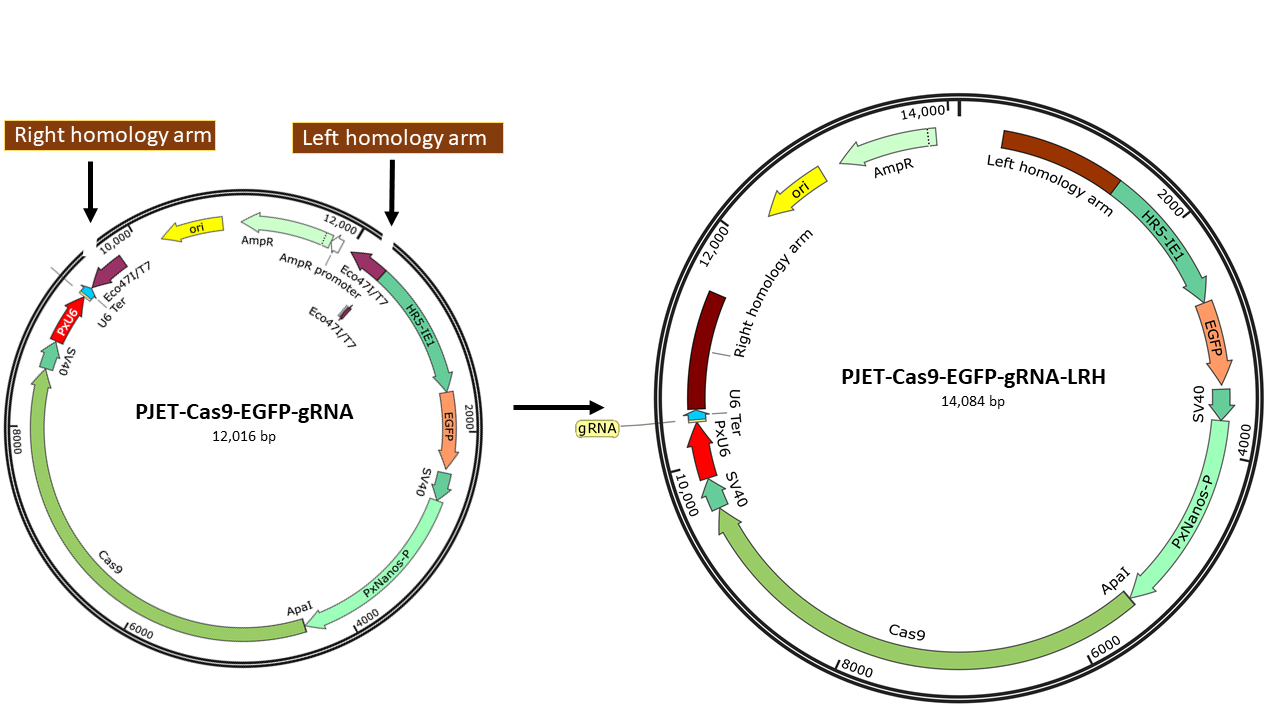


Figure S4 Schematic representation for assembling the left and right homology arms into PJET-Cas9-EGFP-gRNA vector to obtain the full gene drive cassette (PJET-Cas9-EGFP-gRNA -LRH).


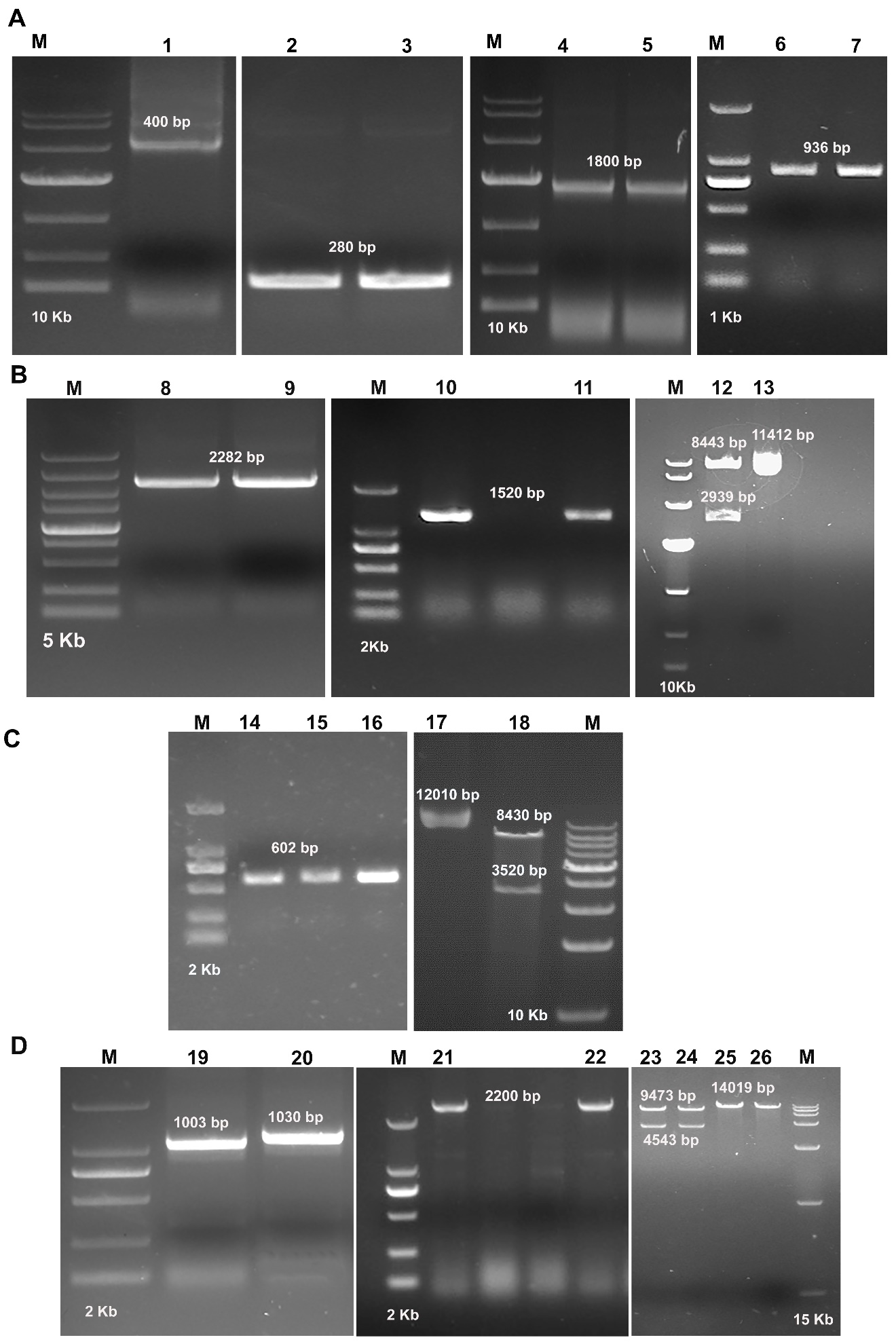


Figure S5 Gel electrophoresis images for the constructions of gene drive cassette (PJET-Cas9-EGFP-gRNA-LRH). A represents the gel electrophoresis images for PCR amplification and insertion of three fragments into PJET 1.2 vector to obtain the PJET-Cas9 vector. Lane M for marker; 1 for the PCR amplified products of Cas9; 2, 3, for the PCR amplified products of Sv40; 4, 5 for PCR amplified product of PxNanosP; and 6,7 for colony PCR confirmation of successful insertion of three fragments into PJET 1.2 vector. B reprsents Gel electrophoreses images for PCR amplification and insertion of HR5IE1-EGFP fragment to PJET-Cas9. Lane 8, 9 for PCR amplified products of HR5IE1-EGFP; 10, 11 for colony PCR confirmation of successfully insertion; lane 12 for the double digestion of vector PJET-Cas9-EGFP and lane 13 for undigested vector. C represents the Gel electrophoreses images for PCR amplification and insertion of PxU6-gRNA fragment to PJET-Cas9-EGFP vector; lane 14, 15, 16 for PCR amplified products of PxU6-gRNA; lane 17 for undigested PJET-Cas9-EGFP-gRNA vector; lane 18 for products of the vector PJET-Cas9-EGFP-gRNA after double digestion. D represents the Gel electrophoreses images for PCR amplification and insertion of LHA and RHA fragments to PJET-Cas9-EGFP-gRNA; lane 19 for PCR amplified products of LHA; lane 20 for PCR amplified products of RHA; lanes 21, 22, for the product of colony PCR; lane 23, 24 for the products PJET-Cas9-EGFP-gRNA-LRH vector after double digestion and lane 25, 26 for undigested PJET-Cas9-EGFP-gRNA-LRH.


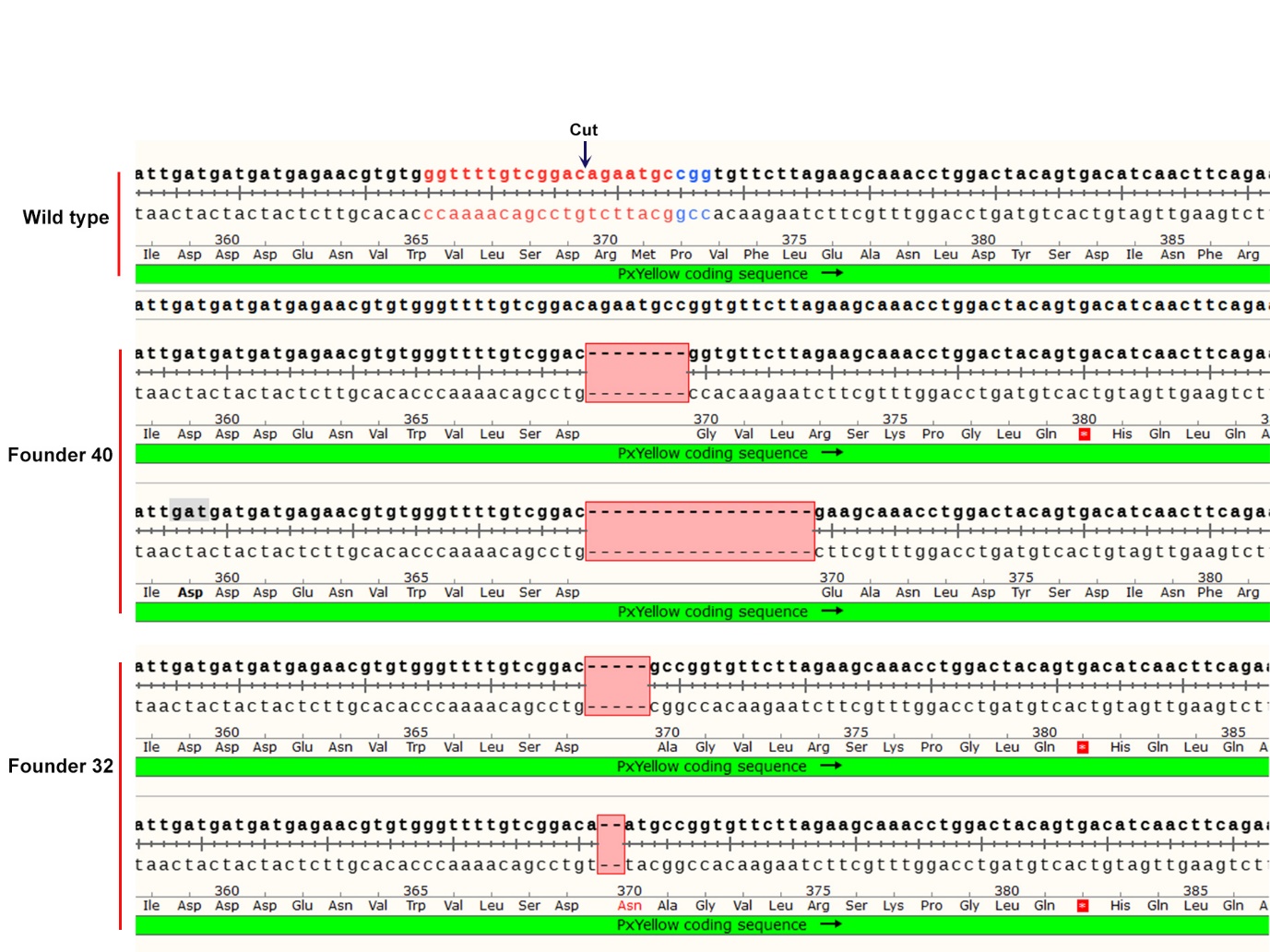


Figure S6 NHEJ-based mutations in *Pxyellow* target site of both founders (40 and 32). The upper sequence is a wild-type reference sequence of the *Pxyellow* target site, and the gRNA sequence is highlighted with red color, and the PAM sequence is shaded with blue color. The black arrow represents the cleavage site inside the gRNA target sequence. The deletion of base pairs in both founders has been shown.
