# Supplementary material for: Development of CRISPR/Cas9-Mediated Gene-Drive Construct Targeting the Phenotypic Gene in Plutella xylostella
Source: Front Physiol. 2022 Jun 29;13:938621. doi: 10.3389/fphys.2022.938621 (PMC9277308; doi:10.3389/fphys.2022.938621)
Supplement: Supplementary file 1 [file DataSheet1.zip › Supplementary files/Supplementary Tables.docx]

**Supplementary Tables**

**Table S1**  **Primers used in this study**

| **Name** | **Sequence** | **Use** |
| --- | --- | --- |
| Yts-F | ATGGCCTTCAAGCTGGTGCTTCT | Amplification of *Pxyellow* |
| Yts-R | TCATGATTCATATACCTCGTAGTTTCC |  |
| sgRNA-F* | TAATACGACTCACTATAGGAGGTTTTGTCGGACAGAATGCCGGTTTTAGAGCTAGAAATAGCAAGTTAAAATAAGGCTAGTCC | gRNA synthesis |
| sgRNA-R | AAAAGCACCGACTCGGTGCCACTTTTTCAAGTTGATAACGGACTAGCCTTATTTTAACTTGCTATTTCTAGCTCTAAAAC |  |
| gRTS-F | AATCGACCAGAACGGCATTG | PCR-based genotyping |
| gRTS-R | TGGTTTCGGTTCGGCGATGC |  |
| Nanos-F | TAGTGACGTCTCCTGGAGTG | Amplification of *PxNanosP* |
| Nanos-R | TTTGTAAAAAGTTAAAATAAACA |  |
| Nanos/Cas9-F | *CTTCCGGATGGCTCGAGTTTTTCAGCAAGAT***CCTCGAGG**TAGTGACGTCTCCTGGA | Assembling of *PxNanosP*, *Cas9* and *Sv40* fragments |
| Nanos/Cas9-R | *TATCGAGCCCAATGGAGTACTTCTTGTCCAT***GGGCCC**TTTGTAAAAAGTTAAAATAAACA |  |
| Cas9-F | *GATTGCATTGTTTATTTTAACTTTTTACAAA***GGGCCC**ATGGACAAGAAGTACTCCATTGG |  |
| Cas9-R | *ATTATGATCAGTTATCTAGATCCGGTGGATC*TCACACCTTCCTCTTCTTCTTGGGGTCAG |  |
| Sv40-F | *GCTGACCCCAAGAAGAAGAGGAAGGTGTGA*GATCCACCGGATCTAGATAACTGATCATAA |  |
| Sv40-F | *AGAATATTGTAGGAGATCTTCTAGAAAGATTC****ACCGGT***CTCGCGTTAAGATACATTGATG |  |
| C.PCR Cas9-F | AATACGGCGGATTCGATTCTCC | Colony PCR confirmation |
| C.PCR Sv40-R | TTATGTTTCAGGTTCAGGGGGA |  |
| IE1-F | *CGGATGGCTCGAGTTTTTCAGCAAGAT***ACTAGT**CATTGCTTGTCATTTATTAATTTGG | Insertion of HR5IE1-EFGP |
| IE1-R | *GTGTTACCTCACTCCAGGAGACGTCACTA*TCTTAATTAACTCGCGTTAAGATACAT |  |
| C.PCR IE1-F | CGATAACCGCGTTGGTTTTAGAG | Colony PCR confirmation |
| C.PCR Sv40-R | TTATGTTTCAGGTTCAGGGGGA |  |
| U6-F | AGGAGTCCCATACCTAACAGTAAG | Link to *PxU6* and gRNA |
| U6-R | GCATTCTGTCCGACAAAACCACTAATGAGGGATTCAAATT |  |
| U6-sacff-R | AAAAAAGCACCGACTCGGTGCCACTTTTTCAAGTTGATAACGGACTAGCCTTATTTTAACTTGCTATTTCTAGCTCTAAAACGCATTCTGTCCGACAAAACC |  |
| gRNA-F | *ACTCATCAATGTATCTTAACGCGAG***ACCGGT**AGGAGTCCCATACCTAACAGTAAG | Assembling of *PxU6*-gRNA |
| gRNA-R | *GGAGATCTTCTAGAAAGATTCC***GCCGGCG**AAAAAAGCACCGACTCGGTGCCACTT |  |
| LH-F | TGAACTACATCCCTCTCGATGCTCC | Amplification of left homology arm |
| LH-R | CACACGTTCTCATCATCATCAATC |  |
| RH-F | TGTTCTTAGAAGCAAACCTGGACT | Amplification of right homology arm |
| RH-R | ACAATATTTGTCCTTGAGTATAGGACG |  |
| LHA-F | *GGATGGCTCGAGTTTTTCAGCAAGATA*TGAACTACATCCCTCTCGATGCTCC | Assembling of left homology arm |
| LHA-R | *TCCAAATTAATAAATGACAAGCAATG***ACTAGT**CACACGTTCTCATCATCATCAATC |  |
| RHA-F | *AAAGTGGCACCGAGTCGGTGCTTTTTT***CGCCGGC**GTGTTCTTAGAAGCAAACCTGGACT | Assembling of left homology arm |
| RHA-R | *TTGTAGGAGATCTTCTAGAAAGATTCCG***CCTCGAGG**TACAATATTTGTCCTTGAGTATAGGACG |  |
| LH.C.PCR-F | CCTGTCCTTACGCCCTTAAC | Colony PCR confirmation |
| LH.C.PCR-R | TCTACTCGTAAAGCGAGTTCAG |  |
| RH.PCR-F | ATACGACCTGCAAGGGAACCT | Colony PCR confirmation |
| RH.C.PCR-R | TGGTTTCGGTTCGGCGATGC |  |
| LH conf-F | ATGTGGTAATACGCGACAGG | Confirmation of left homology arm |
| LHC-R | TCTACTCGTAAAGCGAGTTCAG |  |
| RHC-F | TTCAGGTGGGCAACGGAGAC | Confirmation of right homology arm |
| RHC-R | GCGAGTGAAATAGAGGAACCAAC |  |
| gTS-F | AATCGACCAGAACGGCATTG | gRNA target site |
| gTS-R | TAGCGGAAGGTACGTCTGTG |  |
| Red color represents the T7 promoter, Italic sequences are indicated the homology region for assembling the fragments, underline sequences represent gRNA scaffold, Blue color represents the gRNA sequence, bold sequences represent the endonuclease restriction site. | | |

Table S2 G_0_ survival rate after embryo injection

| Attempt | Injected embryo | Survival of G_0_ | Survival rate (%) |
| --- | --- | --- | --- |
| 1^st^ | 540 | 110 | 20.37 |
| 2^nd^ | 1050 | 270 | 25.71 |
| 3^rd^ | 1240 | 290 | 23.38 |
| 4^th^ | 2120 | 448 | 21.13 |
| 5^th^ | 980 | 188 | 19.18 |
| Total | 5930 | 1306 | 22.02 |

Table S3 Crossing of G_0_ with wild type to obtain F_1_ generation

| G_0_ | Wild-type | F_1_ |
| --- | --- | --- |
| 90 males | 180 females | 17700 |
| 98 females | 196 males | 10500 |
| Total | | 28200 |
